# Nanoparticle Retinoic Acid-Inducible Gene I Agonist for Cancer Immunotherapy

**DOI:** 10.1021/acsnano.3c06225

**Published:** 2024-04-23

**Authors:** Lihong Wang-Bishop, Mohamed Wehbe, Lucinda E. Pastora, Jinming Yang, Blaise R. Kimmel, Kyle M. Garland, Kyle W. Becker, Carcia S. Carson, Eric W. Roth, Katherine N. Gibson-Corley, David Ulkoski, Venkata Krishnamurthy, Olga Fedorova, Ann Richmond, Anna Marie Pyle, John T. Wilson

**Affiliations:** † Department of Chemical and Biomolecular Engineering, Vanderbilt University, Nashville, Tennessee 37212, United States; ‡ Department of Pharmacology, Vanderbilt University Medical Center, Nashville, Tennessee 37232, United States; § Department of Veterans Affairs, Tennessee Valley Healthcare System, Nashville, Tennessee 37212, United States; ∥ Department of Biomedical Engineering, Vanderbilt University, Nashville, Tennessee 37212, United States; ⊥ Northwestern University Atomic and Nanoscale Characterization Experimental (NUANCE) Center, Northwestern University, Evanston, Illinois 60208, United States; # Department of Pathology, Microbiology, and Immunology, Vanderbilt University Medical Center, Nashville, Tennessee 37232, United States; ∇ Department of Medicine, Vanderbilt University Medical Center, Nashville, Tennessee 37232, United States; ○ Advanced Drug Delivery, Pharmaceutical Sciences, R&D, AstraZeneca, Boston, Massachusetts 02451, United States; ◆ Department of Molecular, Cellular and Developmental Biology, Yale University, New Haven, Connecticut 06520, United States; ¶ Howard Hughes Medical Institute, Chevy Chase, Maryland 20815, United States; & Department of Chemistry, Yale University, New Haven, Connecticut 06520, United States; ● Vanderbilt Institute of Chemical Biology, Vanderbilt University, Nashville, Tennessee 37212, United States; ◊ Vanderbilt Institute of Nanoscale Science and Engineering, Vanderbilt University, Nashville, Tennessee 37212, United States; ▲ Vanderbilt Institute for Infection, Immunology, and Inflammation, Vanderbilt University, Nashville, Tennessee 37212, United States; □ Vanderbilt Center for Immunobiology, Vanderbilt University Medical Center, Nashville, Tennessee 37232, United States; ^ Vanderbilt Ingram Cancer Center, Nashville, Tennessee 37232, United States

**Keywords:** retinoic acid-inducible gene I, innate immunity, lipid nanoparticle, immunotherapy, immune checkpoint blockade

## Abstract

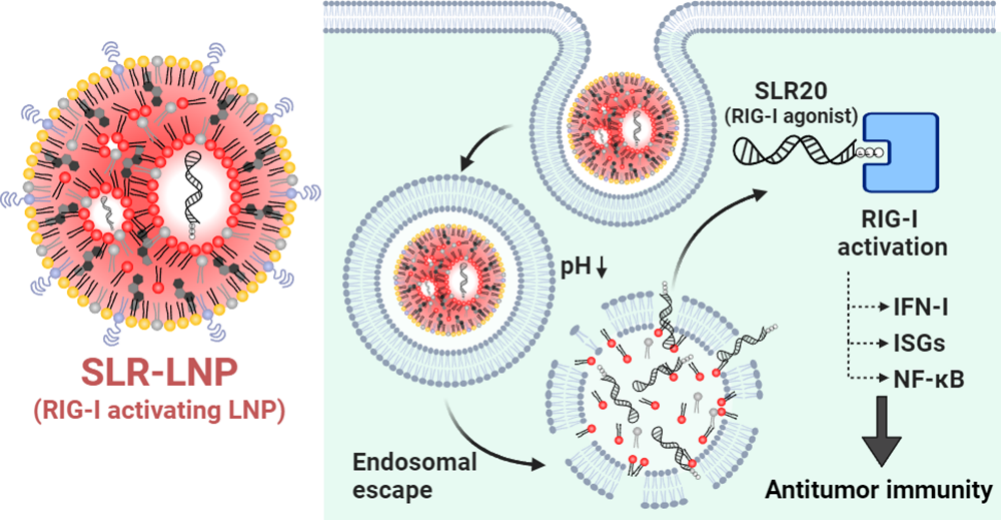

Pharmacological activation of the retinoic acid-inducible gene I (RIG-I) pathway holds promise for increasing tumor immunogenicity and improving the response to immune checkpoint inhibitors (ICIs). However, the potency and clinical efficacy of 5′-triphosphate RNA (3pRNA) agonists of RIG-I are hindered by multiple pharmacological barriers, including poor pharmacokinetics, nuclease degradation, and inefficient delivery to the cytosol where RIG-I is localized. Here, we address these challenges through the design and evaluation of ionizable lipid nanoparticles (LNPs) for the delivery of 3p-modified stem-loop RNAs (SLRs). Packaging of SLRs into LNPs (SLR-LNPs) yielded surface charge-neutral nanoparticles with a size of ∼100 nm that activated RIG-I signaling in vitro and in vivo. SLR-LNPs were safely administered to mice via both intratumoral and intravenous routes, resulting in RIG-I activation in the tumor microenvironment (TME) and the inhibition of tumor growth in mouse models of poorly immunogenic melanoma and breast cancer. Significantly, we found that systemic administration of SLR-LNPs reprogrammed the breast TME to enhance the infiltration of CD8^+^ and CD4^+^ T cells with antitumor function, resulting in enhanced response to αPD-1 ICI in an orthotopic EO771 model of triple-negative breast cancer. Therapeutic efficacy was further demonstrated in a metastatic B16.F10 melanoma model, with systemically administered SLR-LNPs significantly reducing lung metastatic burden compared to combined αPD-1 + αCTLA-4 ICI. Collectively, these studies have established SLR-LNPs as a translationally promising immunotherapeutic nanomedicine for potent and selective activation of RIG-I with the potential to enhance response to ICIs and other immunotherapeutic modalities.

## Introduction

Immunotherapy has revolutionized the treatment of an increasing diversity of tumor types, resulting in robust and durable responses for some patients.^1^ However, it is now well-recognized that, for most cancer types, only a minority of patients respond to currently approved immune checkpoint inhibitors (ICIs) that target CTLA-4 and PD-1/PD-L1.^2^ While resistance to ICIs is multifaceted, for many cancer types, the response to ICI correlates with an immunogenic (“hot”) tumor microenvironment (TME) that is infiltrated with tumor antigen-specific CD8^+^ cytotoxic T cells that are reactivated by immune checkpoint blockade (ICB).^2,3^ However, accumulating data indicate that many patients, perhaps the majority, have immunologically “cold” tumors with low T-cell infiltration that instead have a high density of immunosuppressive cells that inhibit antitumor immunity. This has motivated widespread investigation into the development of therapeutics that reprogram the TME to increase the number and function of tumor-infiltrating T cells that can be reactivated in response to ICIs.^3−5^

Innate immunity plays a critical role in the detection and elimination of cancers.^6^ The innate immune system employs pattern recognition receptors (PRRs)—a network of molecular sensors that detect distinctive features of pathogens or damaged tissue (i.e., “danger signals”)—to trigger inflammatory responses that are critical to the recruitment of immune cell populations to sites of infection, tissue injury, and malignancy.^6,7^ Retinoic acid-inducible gene I (RIG-I) (also known as DDX58) is an important PRR for sensing RNA viruses^8^ via recognition of short, double-stranded RNA with a triphosphate group (3p) on the 5′ end (3pRNA).^9,10^ The 3p group acts as a “tag” that allows RIG-I to discriminate between 3pRNA and other cytosolic RNAs (e.g., mRNA, miRNA, etc.) with high selectivity. RIG-I activation generates a multifaceted inflammatory response resulting in the production of type-I interferons (IFN-I), interferon-stimulated genes (ISGs), T-cell chemokines (e.g., CXCL-9, 10), and proinflammatory cytokines, which cooperate to exert direct and broad-spectrum antiviral functions while also augmenting and shaping the subsequent adaptive immune response.^11−13^ Evidence is also emerging that RIG-I can detect self-RNA derived from aberrantly expressed endogenous retroviral elements dispersed within the human genome or mislocalized mitochondrial RNA in the cytosol and, hence, may also have an important role in promoting endogenous immunity against cancer.^14,15^ Indeed, RIG-I signaling in cancer cells has been shown to dictate responsiveness to anti-CTLA-4 ICB in tumor-bearing mice, consistent with an association between RIG-I expression level, T-cell infiltration, and survival in patients with melanoma.^16^ Such links between RIG-I and endogenous cancer immune surveillance motivate the development of RIG-I agonists as cancer immunotherapies.

While promising as an immunotherapy agent, 3pRNA RIG-I agonists face multiple barriers to therapeutic efficacy that are shared with many oligonucleotide therapies, including a short plasma half-life (i.e., minutes), high susceptibility to nuclease degradation, inefficient intracellular delivery, and, critically, degradation in lysosomes with minimal delivery to the cytosol where RIG-I is located.^17,18^ In considering this drug delivery challenge, we postulated that clinically advanced lipid nanoparticle (LNP) technology could be harnessed to overcome barriers to 3pRNA delivery, thereby facilitating the pharmacological activation of RIG-I as a cancer immunotherapy. LNPs have been widely employed for the delivery of diverse types of nucleic acid therapeutics (e.g., mRNA, siRNA, DNA).^19,20^ Their capacity to efficiently package and facilitate the cytosolic delivery of drug cargo is vital to the success of several FDA-approved LNP-based nanomedicines, including the Moderna and Pfizer-BioNTech mRNA COVID-19 vaccines.^21^ However, LNP formulations of 3pRNA have not yet been explored for the immunotherapeutic activation of RIG-I.

Here, we describe the design and preclinical evaluation of a nanoparticle RIG-I agonist for cancer immunotherapy based on a simple yet highly effective approach. We leveraged an ionizable lipid that is already used in an FDA-approved siRNA therapeutic^22^ to package a 3p-modified, stem-loop RNA (SLR) that we have engineered to be a molecularly defined, selective, and high-affinity RIG-I agonist.^11^ We found that SLR-loaded LNPs (SLR-LNP) inhibited tumor growth and increased the response to ICIs in poorly immunogenic, orthotopic mouse models of breast cancer and melanoma. Importantly, whereas most previous reports^13,23,24^ and early-stage clinical trials (e.g., NCT03739138)^25^ have relied on intratumoral (IT) injection of 3pRNA complexed to the cationic transfection agent jetPEI, SLR-LNPs could be safely administered systemically via intravenous injection, resulting a nearly complete elimination of lung metastases in mice with ICI-resistant metastatic melanoma. Collectively, our studies have yielded among the most potent and effective strategies for pharmacological RIG-I activation described to date and have identified LNPs as a previously unexplored and translationally advanced nanotechnology platform for harnessing the potential of RIG-I in cancer immunotherapy.

## Results

### Lipid Nanoparticle Delivery of SLR Potently Activates RIG-I Signaling

**Figure 1:**
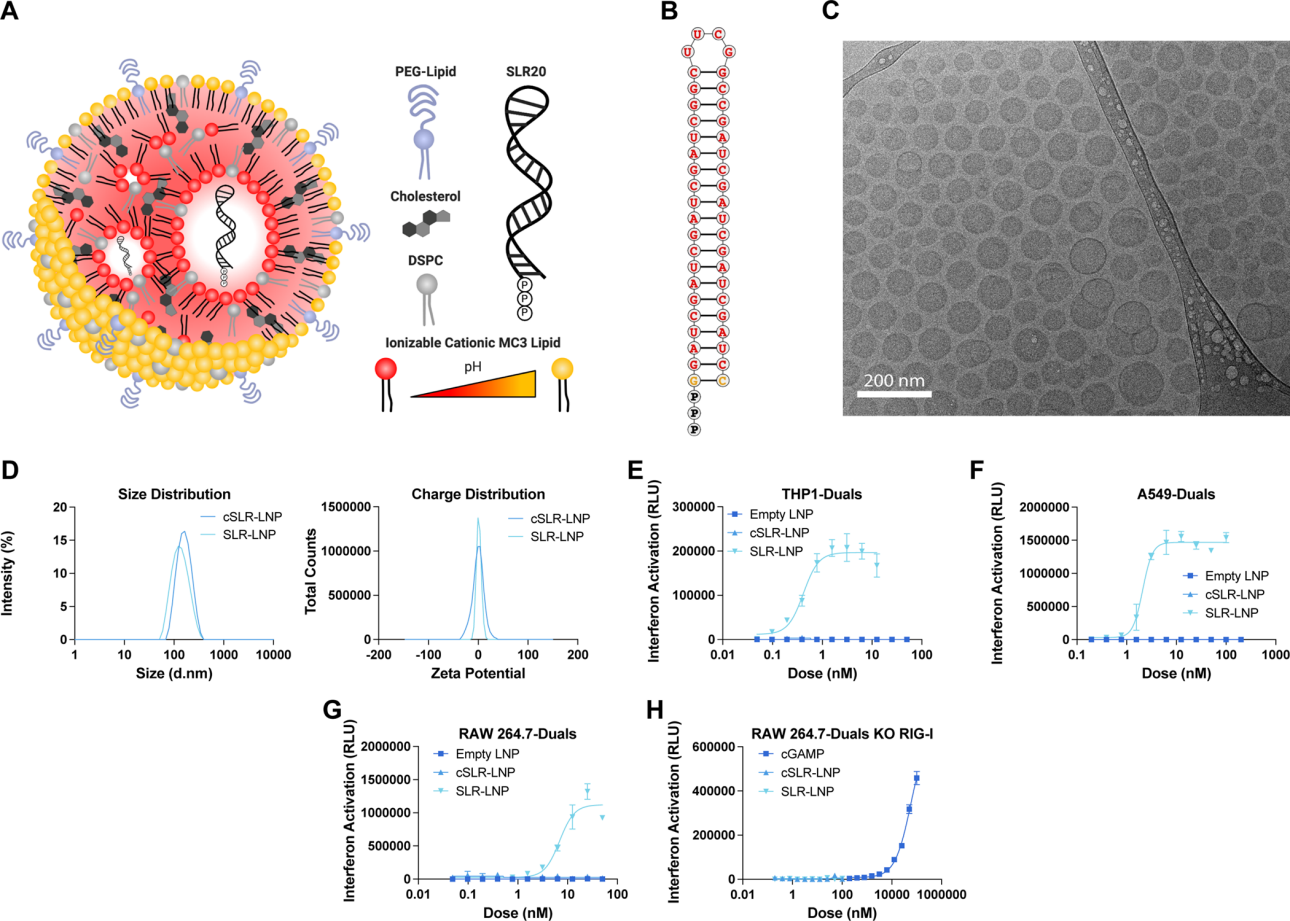
Delivery of SLR20 with lipid nanoparticles potently activates RIG-I. (A) Schematic of SLR-LNP composition and structure. (B) Structure and sequence of SLR20. (C) CryoEM image of SLR-LNP. (D) Nanoparticle size distribution measured by dynamic light scattering and zeta potential at pH 7.4 of LNPs loaded with SLR20 and negative control SLR (cSLR). Dose–response curves for type-I IFN (IFN-I) elicited by indicated LNP formulations in (E) THP1, (F) A549, and (G) RAW264.7 cells were obtained with an IFN regulatory factor (IRF)-inducible reporter construct. (H) Dose–response curve of the IFN-I response elicited by indicated LNP formulation or the STING agonist cGAMP (positive control) in RAW264.7 KO-RIG-I cells with an IFN regulatory factor (IRF)-inducible luciferase reporter.

LNPs consist of several types of lipids, including “helper” lipids that contribute to structure and delivery efficiency, lipids modified with poly(ethylene glycol) (PEG-lipid) to confer colloidal stability and blood compatibility, and, importantly, ionizable lipids that facilitate packaging of RNA cargo via electrostatic interactions and promote the delivery of RNA into the cytoplasm following endocytosis and endosomal acidification.^19,20^ While an ever-expanding number of ionizable lipids are being developed, few are currently approved as components of therapeutics that are administered systemically (i.e., intravenously) in humans.^26^ Therefore, we selected DLin-MC3-DMA (MC3), a component in the FDA-approved, siRNA therapeutic ONPATTRO (patisiran)^22,26^ as a clinically relevant ionizable lipid for our design (Figure 1A). To confer colloidal stability and improve circulation half-life, 3.5% PEGylated lipid (DMG-PEG2000) was used in the formulation as well as 7.5% cholesterol and 31.5% DSPC (1,2-distearoyl-*sn*-glycero-3-phosphocholine) as helper lipids. We increased the amount of PEGylated lipid relative to that used in the patisiran formulation (3.5 vs 1.5%) based on previous work demonstrating that increased PEGylation can increase colloidal and serum stability.^27^ Critical to our design, we also employed a well-defined, high-affinity, stem-loop 3pRNA (SLR) ligand for RIG-I that we have previously leveraged for potent and specific pharmacological activation of RIG-I in mice.^11,17^ SLRs are synthesized using solid-phase nucleic acid synthesis methods, enabling high yield and purity of molecularly defined and potent RIG-I agonists with advantages over double-stranded 3pRNA synthesized via in vitro transcription, which has been primarily utilized. Here, we used SLR20, a single-stranded 44-mer that folds into a stem-loop structure with a 20-base pair stem and a four-nucleotide loop (Figure 1B).

Mixing of lipids and SLR20 in citrate buffer (pH 3) at a nitrogen:phosphate (N:P) ratio of 4.8:1 resulted in the assembly of uniform, spherical SLR-LNP with near 100% RNA encapsulation efficiency, a diameter of ∼100 nm, and a neutral zeta potential (Figure 1C,D). The immunostimulatory activity of SLR-LNP was evaluated in a series of type-I interferon (IFN-I) reporter cell lines, with dose–response studies yielding EC_50_ values in the 1 to 10 nM range, depending on cell types (Figure 1E–H). Importantly, empty LNPs and LNPs loaded with an analogous negative control SLR (cSLR) that lacked the 3p moiety and instead displayed a 5′-hydroxyl group did not induce an IFN-I response. Using RIG-I knockout reporter cells, we also validated that the IFN-I response induced by SLR-LNPs was dependent on RIG-I (Figure 1H). We also tested the activity of SLR-LNPs in primary murine bone marrow-derived dendritic cells (BMDCs), finding that SLR-LNPs, but not empty LNP and cSLR-LNP controls, stimulated expression of IFN-I (*Ifnb1*), interferon-stimulated genes (ISGs) (*Cxcl9*, *Cxcl10*), and Th1 cytokines (*Tnfa*, *Il12*) (Figure S1A) and increased surface expression of the dendritic cell (DC) activation and maturation markers CD80, CD86, and MHC-II (Figure S1B). Finally, since we, and others, have demonstrated that RIG-I activation in cancer cells can be important to therapeutic responses,^16,28,29^ we also tested the activity of SLR-LNPs in B16.F10 melanoma and EO771 breast cancer cells, again demonstrating that SLR-LNPs increased expression of cytokines associated with RIG-I activation relative to controls (Figure S1C,D). Hence, LNPs provide a facile strategy for the efficient packaging and intracellular delivery of SLR20, yielding an immunostimulatory nanoparticle with broad potential clinical utility.

### SLR-LNPs Stimulate Antitumor Innate Immunity

**Figure 2:**
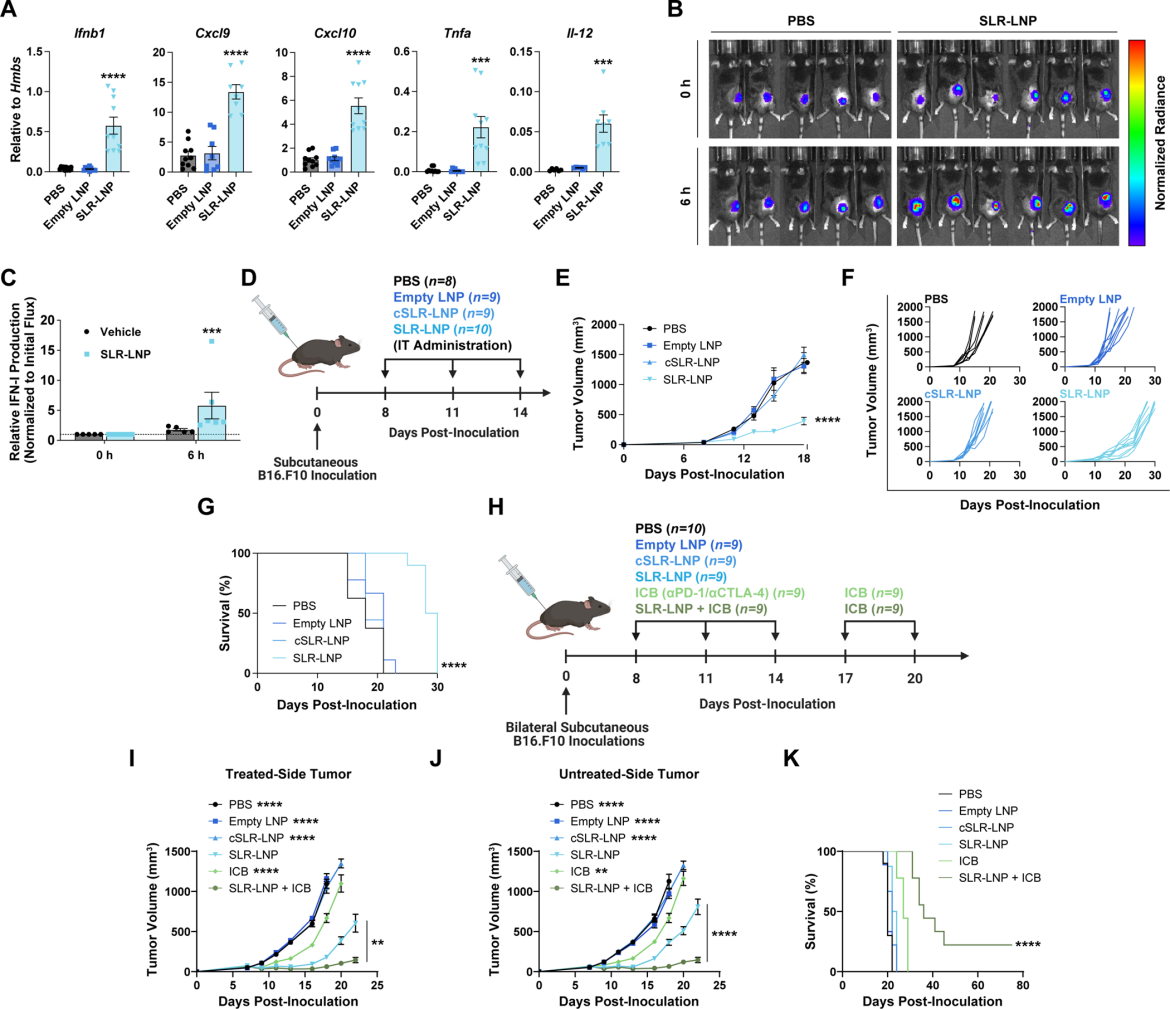
IT administration of SLR-LNPs activates RIG-I in the TME to inhibit local and distal tumor growth. (A) RT-qPCR analysis of B16.F10 tumors after IT injection of a single dose of either PBS, empty LNP, or SLR-LNP (*n* = 10 mice per group, ***P ≤ 0.001; ****P ≤ 0.0001 vs PBS). (B) Representative IVIS luminescence images of mice with B16.F10 IFN-LUC tumors following a single IT injection of either PBS or SLR-LNP. (C) Fold-change (over *t* = 0 h) in luminescence of B16.F10 IFN-LUC tumors 6 h following IT administration of PBS or SLR-LNP (*n* = 6 mice per group; ***P ≤ 0.001 by paired *t* test). (D) Schematic of B16.F10 melanoma tumor inoculation and treatment schedule. (E) Tumor growth curves (****P ≤ 0.0001 compared to PBS at day 18), (F) spider plots, and (G) Kaplan–Meier survival curves (****P ≤ 0.0001 compared to PBS control by log-rank test) of mice with B16.F10 tumors treated as indicated (*n* = 8–10 mice per group). (H) Schematic of two tumor B16.F10 melanoma models and treatment schedule. Tumor growth curves of (I) treated-side tumors (comparisons indicated in legend: ****P ≤ 0.0001 compared to SLR-LNP and SLR-LNP + ICB on day 18; comparisons indicated on the graph: **P ≤ 0.01 between SLR-LNP and SLR-LNP + ICB at day 22 by unpaired *t* test) and (J) untreated-side tumors (comparisons indicated in legend: **P ≤ 0.01; ****P ≤ 0.0001 compared to SLR-LNP and SLR-LNP + ICB on day 18; comparisons indicated on graph: ****P ≤ 0.0001 between SLR-LNP and SLR-LNP + ICB at day 22 by unpaired *t* test). (K) Kaplan–Meier survival curves (****P ≤ 0.001 compared to PBS control by log-rank test) of mice with two B16.F10 tumors treated as indicated (*n* = 9–10 mice per group). All data are presented as mean ± SEM, and P values are determined by one-way ANOVA with post hoc Tukey’s correction for multiple comparisons unless otherwise stated.

We evaluated the in vivo activity of SLR-LNPs in a weakly immunogenic B16.F10 melanoma model that is nonresponsive to ICB, first using the IT administration route that has been most commonly employed for evaluation of RIG-I agonists,^16,24^ including in recent clinical trials.^25^ Consistent with in vitro data, IT injection of SLR-LNPs promoted expression of proinflammatory, antitumor cytokines (*Ifnb1*, *Tnfa*, and *Il12*) as well as *Cxcl9* and *Cxcl10*, important chemokines for directing T-cell infiltration (Figure 2A). We also tested the immunostimulatory activity of SLR-LNP in a melanoma model in which B16.F10 cells express an IFN-inducible luciferase reporter, demonstrating that IT administration of SLR-LNPs increases IFN signaling in the tumor cell compartment (Figure 2B, C).

We next evaluated the effect of SLR-LNPs on tumor growth using the B16.F10 melanoma model, first employing an IT administration route that is used clinically in melanoma patients receiving oncolytic virus therapy (e.g., T-VEC).^30^ We found that intralesional injection of SLR-LNPs inhibited tumor growth, resulting in an increase in survival time, whereas empty LNPs and cSLR-LNPs had no impact on tumor growth inhibition relative to that of vehicle (phosphate-buffered saline (PBS))-treated mice (Figure 2D–G). We also tested therapeutic efficacy in a B16.F10 model in which two tumors were established on opposite flanks, and only one of the tumors (treated) was injected with SLR-LNPs beginning on day 8 when the total tumor volume was ∼100 mm^3^ (Figure 2H). We found that IT administration of SLR-LNP inhibited the growth of the treated tumor (Figure 2I) but also reduced the growth of the distal (untreated tumor) (Figure 2J). As was observed in the single tumor study, empty LNPs and cSLR-LNP had no effect on tumor growth inhibition in this dual-tumor model, indicating that the antitumor response is RIG-I-dependent. We also evaluated SLR-LNPs in combination with αPD-1 and αCTLA-4 ICIs, which are approved for the treatment of metastatic melanoma. As expected in this model, the αPD-1 + αCTLA-4 ICB had little effect on tumor growth but enhanced the efficacy of SLR-LNPs in inhibiting both primary and distal tumor growth and increasing overall survival (Figure 2I–K). These studies demonstrate that IT administration of SLR-LNPs can inhibit both treated and distal tumor growth and increase response to ICIs approved in melanoma. While other materials (e.g., jetPEI) have been employed for local delivery of RIG-I agonists,^16,24^ it is notable that LNPs have now been locally administered via intramuscular injection to millions of people receiving COVID-19 mRNA vaccines, which may accelerate the translation of SLR-LNPs for intralesional therapy.

### Systemic Administration of SLR-LNPs Inhibits Tumor Growth

**Figure 3:**
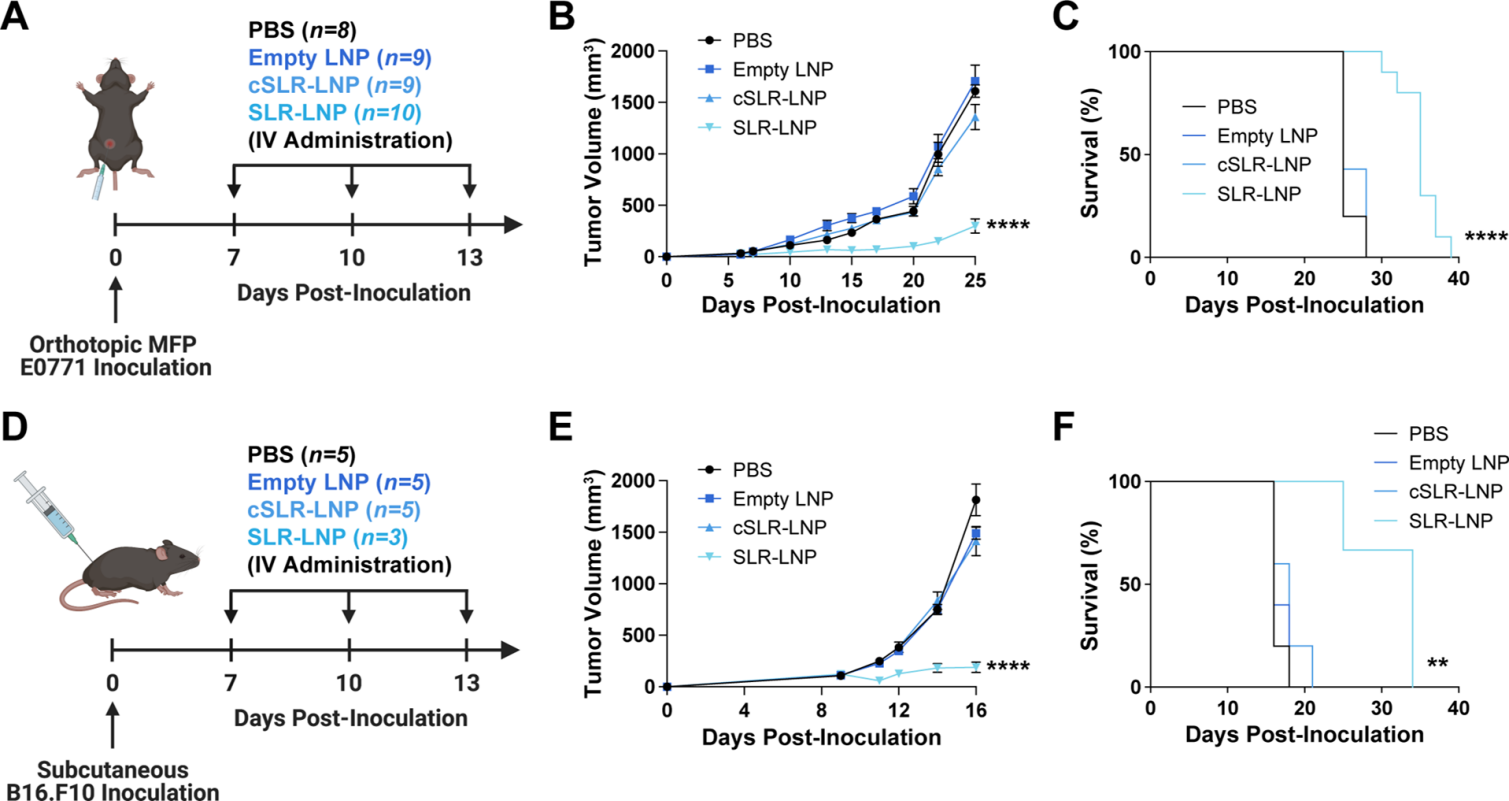
Systemic administration of SLR-LNPs inhibits tumor growth. (A) Schematic of EO771 breast tumor inoculation and treatment timeline. (B) Tumor growth curves (*n* = 8–10 mice per group, ****P ≤ 0.0001 compared to PBS on day 25) and (C) Kaplan–Meier survival curves (**P < 0.01 compared to PBS control by log-rank test) of mice with EO771 tumors treated as indicated (*n* = 8–10 mice per group). (D) Schematic of B16.F10 melanoma and treatment schedule. (E) Tumor growth curves (****P ≤ 0.0001 compared to all other groups on day 16) and (F) Kaplan–Meier survival curves (****P ≤ 0.001 compared to PBS control by log-rank test) of mice with B16.F10 tumors treated as indicated (*n* = 3–5 mice/group). All data are presented as mean ± SEM, and *P* values are determined by one-way ANOVA with post hoc Tukey’s correction for multiple comparisons unless otherwise stated.

While SLR-LNPs hold promise as an intralesional therapy, IT administration may not be possible or practical for many patients and/or cancer types.^31^ Therefore, we next focused our investigations on the larger challenge of achieving the safe and effective systemic administration of RIG-I agonists for cancer immunotherapy. Our group has recently identified RIG-I activation as a potentially promising target for enhancing immunotherapy responses in triple-negative breast cancer (TNBC).^28^ Therefore, we evaluated the efficacy of systemically administered SLR-LNPs in an orthotopic EO771 breast cancer model. We first administered SLR-LNPs or control formulations intravenously at a dose of 10 μg SLR (∼0.5 mg/kg) three times, spaced 3 days apart, and monitored tumor volume (Figure 3A). SLR-LNPs significantly inhibited tumor growth and increased survival time, whereas empty LNPs and cSLR-LNPs had no effect (Figure 3B, C), further demonstrating the importance of RIG-I activation in mediating a therapeutic benefit. We further tested the efficacy of intravenously administered SLR-LNPs in the B16.F10 model, again observing the inhibition of tumor growth and extended survival time (Figure 3D–F).

Importantly, we found that this therapeutic regimen was well tolerated, with mice exhibiting only mild (∼5%) and transient weight loss in the immediate posttreatment period (Figure S2A,B). Consistent with administration of other innate immune agonists, including those that have advanced into the clinic,^18,32,33^ elevated serum cytokine levels were observed 6 h following administration but were insignificant from the background by 24 h (Figure S2C,D). Additionally, no changes in levels of serum BUN, ALT, glucose, or AST were observed (Figure S3), indicating that the treatment did not induce significant liver or kidney damage. Red blood cell (RBC) count and hemoglobin (HGB) levels were slightly reduced for all nanoparticle formulations, but no effect on mean corpuscular hemoglobin (MCH) or MCH concentration (MCHC) was noted. Complete blood count (CBC) revealed no differences relative to the vehicle control, except for neutrophils, which were elevated in response to SLR-LNP treatment. Major organs (liver, spleen, kidney, lung, brain, heart, pancreas, and bone marrow (sternum)) were also isolated 24 h following treatment, routinely fixed in 10% neutral buffered formalin, embedded, sectioned, and stained for blinded evaluation by a board-certified veterinary pathologist. No histopathologic abnormalities were observed in the kidneys, lungs, brain, heart, or pancreas. Histological evidence of a slight increase in extramedullary hematopoiesis in the liver and spleen was observed, and an increased ratio of myeloid to erythroid precursor cells in the bone marrow was noted (Figure S4), both of which were likely secondary to elevated proinflammatory cytokine levels and not clinically significant.

### Systemic Administration of SLR-LNPs Reprograms the TME To Enhance T-Cell Infiltration

**Figure 4:**
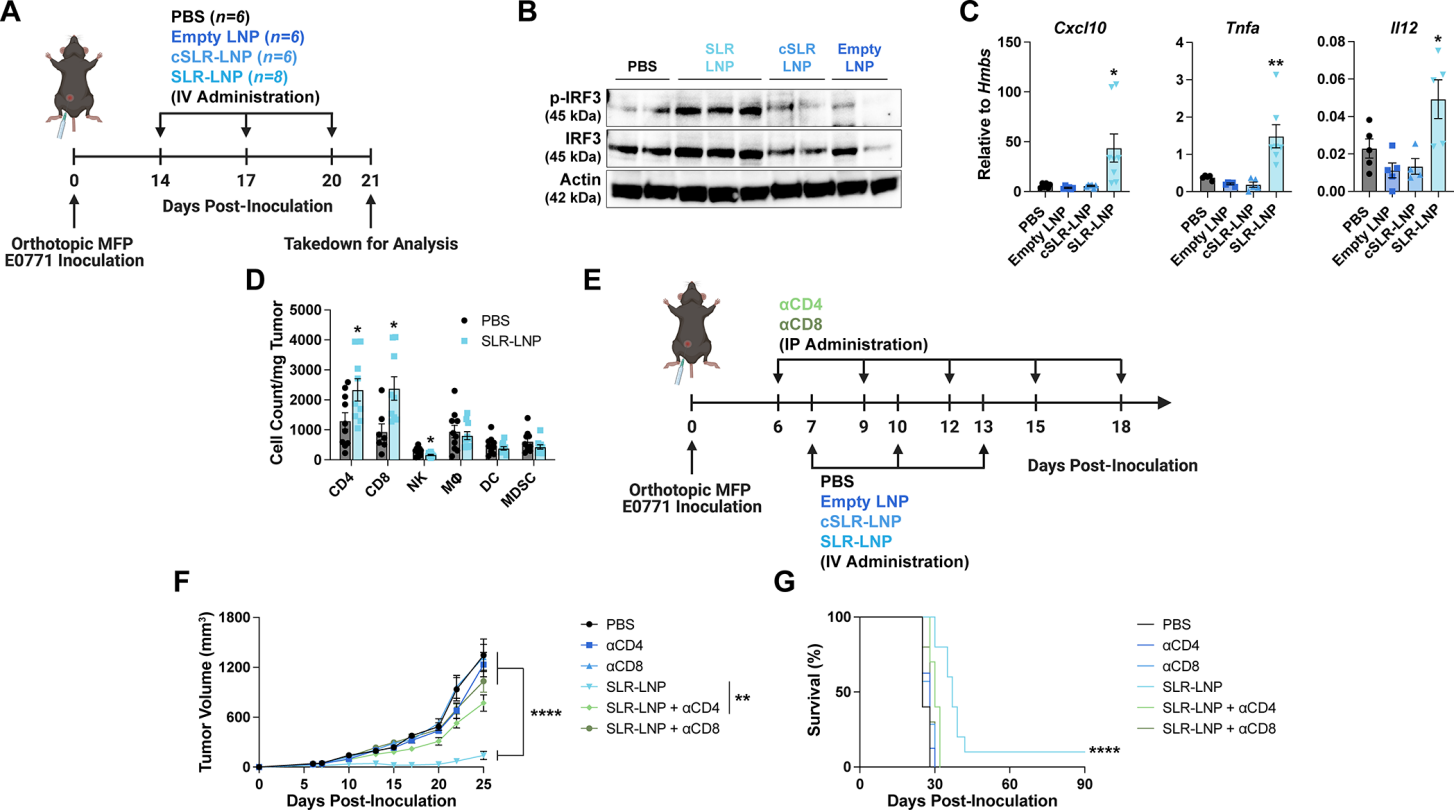
Systemic administration of SLR-LNPs activates RIG-I in the TME to enhance infiltration of CD8^+^ and CD4^+^T cells with antitumor function. (A) Schematic of EO771 breast tumor inoculation, treatment timeline, and analysis time point. (B) Western blot for p-IRF and total IRF in EO771 tumors 24 h after the final administration of SLR-LNPs or indicated controls (*n* = 2–3 mice per group). (C) Analysis of tumor tissue by qRT-PCR 24 h after the final injection of SLR-LNPs or indicated controls (*P ≤ 0.05, **P ≤ 0.01 compared to PBS; *n* = 4–8 mice per group). (D) Flow cytometric quantification of the number (cells/milligram tumor) of immune cells in the EO771 breast TME in response to indicated treatment, including CD8^+^ and CD4^+^ T cells, macrophages (CD11b^+^F4/80^+^), dendritic cells (MHC-II^+^CD11c^+^), NK cells (NK1.1^+^) and MDSCs (CD11b^+^Gr^+^) (*P ≤ 0.05 by paired *t* test; *n* = 10 mice per group). (E) Schematic of EO771 breast tumor inoculation, administration of anti-CD4 (αCD4)- or anti-CD8 (αCD8)-depleting antibodies, and treatment schedule. (F) Tumor growth curves (****P ≤ 0.0001 for SLR-LNP compared to all groups except SLR-LNP + αCD4 and **P ≤ 0.01 for SLR-LNP compared to SLR-LNP + αCD4 at day 25). (G) Kaplan–Meier survival curves (****P < 0.001 compared to PBS control by log-rank test) of mice with EO771 tumors treated as indicated (*n* = 8–10 mice/group). All data are presented as mean ± SEM, and *P* values are determined by one-way ANOVA with post hoc Tukey’s correction for multiple comparisons unless otherwise stated.

Having established a safe and effective regimen for systemic administration of SLR-LNPs, we next evaluated the effects on the TME in the orthotopic EO771 breast cancer model. Tumor tissue was harvested 24 h following the three-dose regimen and processed for analysis by quantitative real-time PCR (qRT-PCR), Western blot, and flow cytometry (Figure 4A). Consistent with RIG-I activation, we observed increased levels of pIRF3 in the TME (Figure 4B) and expression of ISGs and proinflammatory cytokines (Figure 4C) in mice treated with SLR-LNPs, but not empty LNPs or cSLR-LNP formulations. We also observed a significant increase in the number of tumor-infiltrating CD4^+^ and CD8^+^ T cells in response to SLR-LNP treatment, but no significant differences in the number of NK cells, dendritic cells, macrophages, or MDSCs (Figure 4D). Based on these data, we antibody-depleted CD4^+^ and CD8^+^ T cells to elucidate their relative contributions to antitumor efficacy in the EO771 model (Figure 4E–G). Depletion of either T-cell population abrogated therapeutic efficacy, with CD8^+^ T-cell depletion having a slightly larger impact on the antitumor efficacy (Figure 4F,G). Collectively, these studies demonstrate the ability of SLR-LNPs to promote the infiltration of T cells with antitumor function into the TME.

### SLR-LNPs Enhance Response to ICB

**Figure 5:**
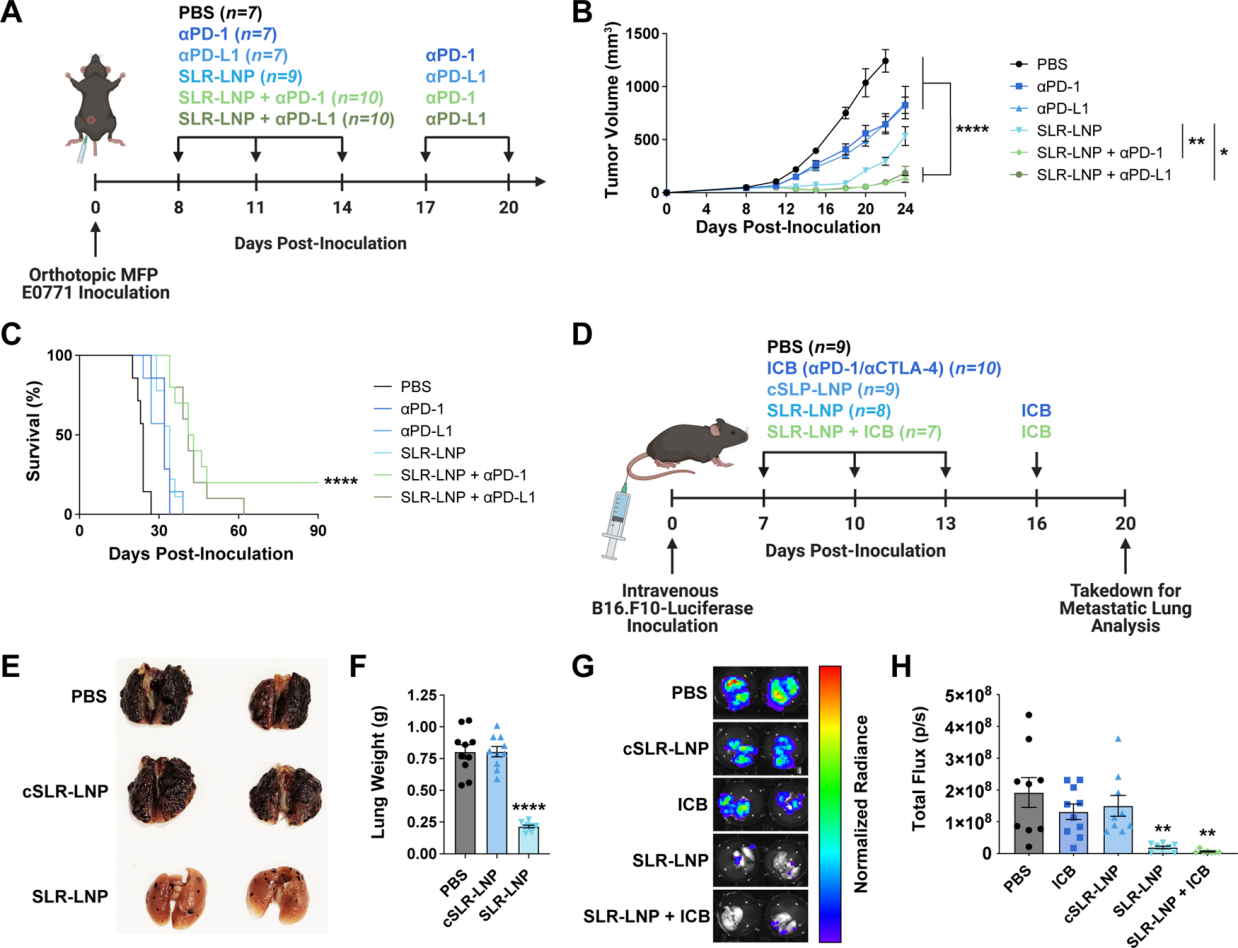
SLR-LNPs enhance response to ICIs. (A) Schematic of EO771 breast tumor inoculation and treatment timeline. (B) Tumor growth curves (****P ≤ 0.0001 for SLR-LNP + αPD-1 and SLR-LNP + αPD-L1 compared to indicated groups, **P ≤ 0.01 for SLR-LNP compared to SLR-LNP + αPD-1, and *P ≤ 0.05 for SLR-LNP compared to SLR-LNP + αPD-L1 at day 24). (C) Kaplan–Meier survival curves (****P < 0.001 compared to PBS control by log-rank test) of mice with EO771 tumors treated as indicated (*n* = 8–10 mice per group). (D) Schematic of B16.F10-Luciferase lung metastasis inoculation, treatment schedule, and analysis time point. (E) Representative images of the lungs from mice treated with PBS, cSLR-LNP, and SLR-LNP. (F) Quantification of lung weight (****P ≤ 0.0001 compared to all other groups). (G) Representative IVIS luminescence images and (H) quantification luminescence of lungs from mice with B16.F10-Luciferase lung metastases (**P ≤ 0.01 compared to PBS; *n* = 10 mice per group). All data are presented as mean ± SEM, and *P* values are determined by one-way ANOVA with post hoc Tukey’s correction for multiple comparisons unless otherwise stated.

Based on the capacity of SLR-LNPs to promote T-cell infiltration into the breast TME, we next evaluated SLR-LNPs in combination with αPD-1 ICI, which is approved for a subset of TNBC patients, who experience a response rate of only ∼20%.^34^ Mice with orthotopic EO771 tumors were treated with SLR-LNPs alone or in combination with anti-PD-1 ICI and tumor volume was monitored (Figure 5A). SLR-LNPs inhibited tumor growth to a greater degree than αPD-1, which exerted only minimal efficacy as monotherapy (Figure 5B), and the combination of SLR-LNPs and αPD-1 further inhibited tumor growth and extended survival, resulting in a 25% (2/8) complete response rate (Figure 5C). We also tested SLR-LNPs in the context of aggressive metastatic melanoma, a setting where systemic administration of RIG-I agonists may be necessary. As a model of lung metastasis, luciferase-expressing B16.F10 cells were injected intravenously to colonize the lung, and mice were subsequently treated with SLR-LNP alone and in combination with αCTLA-4 + αPD-1 ICI (Figure 5D). Mice were euthanized 20 days posttumor inoculation, and lung metastatic burden was evaluated via luminescence and lung mass measurements. Consistent with our other findings, SLR-LNPs, but not cSLR-LNPs, dramatically inhibited tumor formation in the lung, an effect that was further, though not significantly, enhanced with the addition of αCTLA-4 + αPD-1 ICI, which had no effect as a monotherapy in this model (Figure 5E–H). Hence, systemic administration of SLR-LNPs can inhibit tumor growth and metastasis as well as increase response to approved ICIs in multiple poorly immunogenic tumor models.

## Discussion

Identifying agents that remodel the TME from “cold” (i.e., low T-cell infiltration) to “hot” (i.e., T cell inflamed) has emerged as a promising approach for reversing resistance to ICIs.^5,6,35,36^ RIG-I has high potential as a target for increasing tumor immunogenicity and improving response to immunotherapy, but major pharmacological barriers limit the activity and efficacy of 3pRNA as a nucleic acid therapeutic. Here, we address this challenge using a facile and translationally ready strategy that leverages advanced LNP technology and a molecularly engineered SLR to fabricate an immunotherapeutic nanomedicine for potent activation of RIG-I signaling. We found that SLR-LNPs, administered either intratumorally or intravenously, activated RIG-I in the TME, resulting in enhanced effector T-cell infiltration that inhibited tumor growth and enhanced the response to ICIs in multiple immunologically “cold” tumor models. This represents a different application of LNPs and establishes a foundation for further optimization and the preclinical development of SLR-LNPs for cancer immunotherapy.

Despite the promise of 3pRNA as an immunopotentiator, there has been relatively little investigation into the design and testing of carriers to enhance its efficacy.^17,37−42^ Several groups have employed PLGA-based micro- and nanoparticle formulations for 3pRNA delivery, primarily for vaccine applications.^37,38^ Similarly, Bourquin et al. employed cationized gelatin nanoparticles to improve 3pRNA delivery and demonstrated that this enhanced its activity as a vaccine adjuvant.^42^ Huang and co-workers described 3pRNA-loaded lipid calcium phosphate nanoparticles and demonstrated that systemic administration could inhibit tumor growth in models of pancreatic cancer.^39^ Recently, Peng et al. loaded erythrocyte-derived extracellular vesicles with 3pRNA and demonstrated antitumor efficacy when administered intratumorally or via pulmonary delivery for treatment of lung metastasis.^41^ Our group has described polymeric carriers for 3pRNA delivery by exploiting the combinatorial diversity enabled through the synthesis of polymer and SLR structural libraries.^17,40^ However, recent clinical trials,^25^ and most preclinical studies to date,^11,13,23,24^ have employed the cationic polymeric transfection reagent jetPEI, which electrostatically condenses nucleic acids and facilities their release from the endolysosome.^43^ While PEI-based approaches remain promising, and merit continued development, their clinical translation has been hindered by toxicity concerns, a proclivity for accumulation in the lungs, and a relatively low efficiency for cytosolic delivery via the still debated “proton sponge” mechanism.^44,45^ By contrast, LNPs have rapidly emerged as one of the most versatile platforms for delivery of a diverse array of nucleic acids and are essential to the efficacy of several recently approved nucleic acid therapeutics.^26^ Additionally, LNPs are approved for administration both locally (e.g., as mRNA vaccines) and intravenously (e.g., as siRNA therapeutics), providing a versatile drug carrier for both intralesional therapy and systemic therapy for the treatment of metastatic disease.

Surprisingly, there has been virtually no investigation into the use of LNPs for delivery of 3pRNA, which faces delivery barriers common to other classes of oligonucleotide therapeutics but is distinguished by its immunopharmacological mechanisms of action. Hence, we sought to fill this knowledge and innovation gap by leveraging LNP technology to design and test a nanoscale platform for RIG-I activation. Our selection of the MC3 ionizable lipid was primarily motivated by translational considerations as it is already approved for clinical use and, therefore, represented a logical initial choice for the design of RIG-I activating lipid nanoparticles. However, there is also now a vast toolbox of ionizable lipids available for RNA delivery that vary in headgroup and lipid tail structure and can be leveraged to optimize delivery of a specific nucleic acid cargo.^26^ Further, LNP formulations can be assembled with different types and/or compositions of helper and PEGylated lipids using different fabrication approaches, which can be harnessed to modulate pharmacokinetics and/or to confer tissue- or cell-specific tropism that can be tuned for specific disease applications.^46^ Hence, there is a virtually limitless parameter space for the design of LNPs for the delivery of 3pRNA that can now be explored by building upon the SLR-LNP platform for immunotherapy.

The design of drug carriers for 3pRNA therapeutics will ultimately be driven by an understanding of the pharmacological mechanisms of efficacy and toxicity. Such knowledge remains limited for this class of oligonucleotide therapeutics due, in part, to a dearth of technologies that have been developed and/or tested for 3pRNA administration. In this regard, our investigations provide a preclinical benchmark for evaluating systemically administered RIG-I agonists and their carriers. It is important to recognize that even the most promising nanoparticles, LNPs or otherwise, deliver only a small fraction of their nucleic acid cargo to tumor sites and primarily distribute to the liver and/or spleen, depending on nanoparticle properties and/or composition. Indeed, and unsurprisingly, in a preliminary analysis of SLR-LNP pharmacokinetics and biodistribution, we found that the majority of particles accumulated in the liver and the spleen with modest but evident accumulation in orthotopic EO771 breast tumors following intravenous injection (Figure S5). Accordingly, while systemic administration of SLR-LNPs activates RIG-I in the TME, this also results in a transient elevation of serum cytokines (Figure S2) and activation of an antiviral-like innate immune response in the liver and the spleen (Figure S5), which has also been observed for other promising immunostimulatory nanomedicines.^47−50^ Therefore, an important distinction between 3pRNA and more conventional classes of nucleic acid therapeutics for cancer (e.g., siRNA) is that 3pRNA likely exerts robust therapeutic effects via both local (e.g., within the tumor) and systemic (e.g., splenic) reprogramming of immune cell populations that can initiate and propagate antitumor immunity. Such systemic immunopotentiation also obviates the need to deliver high drug doses to the majority of cancer cells at all tumor sites in the body, an established limitation of virtually all nanoparticle delivery platforms.^51^ In this regard, the discordance between the tumor accumulation and therapeutic activity of SLR-LNPs exemplifies this paradigm shift in nanomedicine and motivates re-evaluation of conventional design criteria for immunostimulatory nanoparticles.

While systemic mobilization of an antitumor immune response may be advantageous, and perhaps even necessary, for the treatment of advanced metastatic disease, the potential of intravenously administrated PRR agonists to induce inflammatory toxicities must be considered.^18^ Therefore, we performed a robust preclinical analysis of toxicity following a therapeutic three-dose regimen and found SLR-LNPs to be well tolerated, with mice experiencing only mild, transient weight loss without evidence of organ pathology or abnormal blood chemistry test results. It is notable that other promising innate immune agonists similarly induce a transient elevation of serum cytokines and weight loss in mice,^50,52^ including nanomedicines and antibody-drug conjugates that have advanced into clinical testing with patients experiencing transient flu-like symptoms or other adverse events that were readily manageable.^33,48^ Nonetheless, the systemic cytokine response triggered by intravenously administered SLR-LNPs may limit the therapeutic window and is perhaps the largest barrier to the clinical translation of SLR-LNPs (and RIG-I agonists, more generally). Therefore, an important future direction will be to engineer SLR-LNPs to further enrich RIG-I activation in the TME while minimizing systemic inflammatory responses. Toward this end, our group has described the design of 3pRNA prodrugs that employ bulky covalently linked macromolecules (e.g, PEG) to block recognition of 3pRNA by RIG-I until they are removed under a specific environmental stimulus (e.g., enzymes, redox).^53^ Likewise, there is a deep nanomedicine toolbox available for improving cargo delivery to tumor sites, including improving SLR-LNP half-life and/or integration of molecular targeting moieties or “sheddable” coronas, which could be harnessed to expand the therapeutic window of systemically administered RIG-I agonists.

We demonstrate that SLR-LNPs can enhance T-cell infiltration into tumors and that the therapeutic response is T-cell-mediated. While additional investigation is necessary to identify the primary cellular responders to SLR-LNPs and to further examine their immunopharmacological effects on the tumor and secondary lymphoid tissues, their ability to remodel the TME to increase CD8^+^ and CD4^+^ T-cell infiltration also offers exciting opportunities for the further development of combination immune receptors to enhance therapeutic responses. Here, we focused our investigations on combining SLR-LNPs with approved ICIs based on the recent Phase I clinical trials that explored IT injection of 3pRNA combined with anti-PD-1 (pembrolizumab).^25^ However, there is also a strong immunological rationale for combining RIG-I agonists with other approved and experimental therapeutics, including chemotherapy and other immunomodulators. Furthermore, SLR-LNPs increase the infiltration of endogenous T cells into tumors, presenting the possibility of using intravenously administered SLR-LNPs to enhance responses to other T-cell-based immunotherapeutic modalities—including adoptive T-cell transfer, CAR T-cell therapy, and cancer vaccines—where poor tumor infiltration restricts efficacy in solid tumors.^37,54^ Thus, SLR-LNPs should be further investigated in combination with a variety of antitumor regimens because they may have broad immunotherapeutic utility.

## Conclusions

In conclusion, we have described the fabrication, characterization, and preclinical evaluation of a nanoparticle-based immunotherapy that enhances antitumor immunity via activation of the RIG-I pathway. Our design of a nanoparticle RIG-I agonist was inspired by currently approved lipid nanoparticle formulations for other classes of RNA therapeutics, and we leveraged the ionizable lipid DLin-MC3-DMA to package and enhance the intracellular delivery of selective and well-defined 5′-triphosphate SLR RIG-I ligands. We demonstrated that this strategy resulted in potent activation RIG-I signaling in vitro and in vivo, and that SLR-LNPs could be safely administered via both IT and intravenous routes to promote RIG-I activation in the TME, resulting in expression of type-I interferons, proinflammatory cytokines, and chemokines that enhanced the infiltration of CD8^+^ and CD4^+^ T cells with antitumor function. Consequently, SLR-LNPs inhibited tumor growth in an RIG-I-dependent manner in multiple poorly immunogenic solid tumor models and increased therapeutic responses to anti-PD-1 and anti-CTLA-4 ICIs. Collectively, these studies establish LNP-based packaging of SLRs as a translationally promising strategy for generating nanoparticle RIG-I agonists for cancer immunotherapy.

## Materials and Methods

### DLin-MC3-DMA Lipid Synthesis

DLin-MC3-DMA (MC3) was prepared following the method described in WO2010144740 (Example 5, page 140). Detailed synthesis methods are available in the Supporting Information, and characterization by ^1^H NMR, UPLC-ELSD, and mass spectrometry is provided in Figure S6.

### Formulation of SLR-LNPs

SLR20 was synthesized and purified as described previously.^17,24^ LNP formulations of SLR20 were prepared as previously described for formulation of siRNA-loaded LNPs with minor modifications.^55^ Briefly, DLin-MC3-DMA, 1,2-distearoyl-*sn*-glycero-3-phosphocholine (DSPC) (Avanti Polar Lipids), cholesterol (Avanti Polar Lipids), and PEG_2 kDa_-lipid (PEG-DMG) (Avanti Polar Lipids) were solubilized at a molar ratio of 57.5:7.5:31.5:3.5 in ethanol and heated to 65 °C prior to dropwise addition into citrate buffer (0.1 M, pH 3, 25 °C) under constant mixing to a final volume ratio of 1:3 ethanol to citrate buffer. For SLR-containing formulations, SLRs were dissolved in citrate buffer prior to lipid addition at a concentration that resulted in a final SLR weight fraction (w/w) of 0.06 SLR/Dlin-MC3-DMA; for in vivo studies, an SLR weight fraction of 0.1 was used. Homogenous mixing was allowed to occur for at least 1 h at room temperature to ensure nanoparticle formation. The ethanol and citrate were removed via buffer exchange with PBS (pH 7.4; 3 mM Na_2_HPO_4_, 1 mM KH2PO4, 155 mM NaCl) by dialysis using an Amicon Ultra-15 Centrifugal Filter Unit (100 kDa molecular weight cutoff) regenerated cellulose membrane (Millipore) or via tangential flow filtration (Repligen; KrosFlo Research I Peristaltic Pump with MicroKros Hollow Fiber Filter) for larger batches used for mouse studies. Particle size and zeta potential were determined by using a Malvern Zetasizer Nano ZS instrument at room temperature, and each measurement was independently repeated three times for each sample. The amount of encapsulated nucleic acid was quantified by using the Quant-it RiboGreen RNA assay kit (Invitrogen). Briefly, LNPs were disrupted in 2% Triton X-100 in TE buffer; RiboGreen solution was added to these samples, and fluorescence was measured using a plate reader (Synergy H1Multi-Mode Microplate Reader; Biotek). The RNA concentration was then determined by comparing the fluorescence of the LNP samples to that of the SLR20 or SLROH standard curves.

### DLS and Zeta Potential Measurement of LNPs

Size, PDI, and zeta potentials of LNPs were analyzed by using a Malvern Zetasizer at the Vanderbilt Institute for Nanoscale Science and Engineering. For size and PDI measurements, LNPs were diluted in sterile PBS in a 1.5 mL semimicro cuvette. For zeta potential measurements, LNPs were diluted in NaCl (final concentration 10 mM) and were measured in a DTS1070 capillary cell.

### CryoEM of SLR-LNP

Lacey carbon grids (200 mesh) were glow-discharged for 30 s in a Pelco easiGlow glow-discharger (15 mA; chamber pressure of 0.24 mbar). The sample (4 μL) was pipetted onto a grid, blotted for 5 s, and plunge-frozen into liquid ethane using an FEI Vitrobot Mark IV cryo plunge-freezing robot. Grids were then loaded into a Gatan 626.5 cryo transfer holder and imaged at −180 °C in a JEOL 1400 Flash TEM LaB6 emission TEM at 120 kV. Data were collected with Gatan Digital Micrograph software connected to a Gatan OneView 4k camera.

### Cell Culture

B16–F10 cells were purchased from the American Type Culture Collection (ATCC) and RAW-Dual, THP1-Dual, A549-Dual, and RAW-Dual ISG-KO-RIG-I were purchased from InvivoGen. EO771 cells were gifted from Justin Balko (Vanderbilt University Medical Center), luciferase-expressing B16–F10 cells (B16-LUC) were provided by Ann Richmond (Vanderbilt University Medical Center), and ovalbumin-expressing B16–F10 (B16-OVA) cells were gifted from Amanda Lund (New York University School of Medicine). B16–F10 cells expressing an interferon-inducible luciferase reporter were used as described previously.^52^ All cell lines were cultured according to the manufacturer’s specifications. BMDCs were isolated from 6- to 8-week-old C57BL/6 mice and cultured as described previously.^56^ Briefly, bone marrow was flushed from the femurs and tibias of mice using complete BMDC culture medium (RPMI 640 medium supplemented with 100 U/mL penicillin, 100 μg/mL streptomycin, 1 mM sodium pyruvate, 2 mM l-glutamine, 1× nonessential amino acids, 10 mM HEPES, 50 μM β-mercaptoethanol, and 10% heat-inactivated FBS), and the marrow was passed through a 70 μM cell strainer. Strained bone marrow cells were pelleted and resuspended in warm ACK lysis buffer (ThermoFisher) for 5 min and washed with cold PBS. Then, the cells were seeded in 100 × 15 mm Petri dishes in a complete medium supplemented with 20 ng/mL GM-CSF and maintained in a 37 °C incubator supplemented with 5% CO_2_. The culture medium containing GM-CSF was replaced on days 3, 5, and 7. On day 8, the bone marrow cells were confirmed to be >80% BMDCs (CD11c^+^) by flow cytometry.

### Evaluation of Immunostimulatory Activity in ISG Reporter Cells

RAW-Dual, THP1-Dual, A549-Dual, and RAW-Dual ISG-KO-RIG-I cells were seeded at 50,000 cells/well in 100 μL media in clear cell culture-treated 96-well plates (Greiner Bio-One). When adherent cells became ∼80% confluent or suspension cells reached a density of 1.5 × 10^6^ cells/mL, SLR-LNPs or controls were added to wells at 2x concentration in 100 μL media. Supernatant was collected 24 h after treatment, and the Quanti-Luc (Invivogen) assay was used to determine the amount of secreted luciferase per the manufacturer’s instructions. Briefly, 50 μL of Quanti-Luc solution was injected into each well of a white opaque 96-well plate (Greiner Bio-One) containing 20 μL of collected supernatant per well on a Synergy H1 multimode microplate reader, and each well was immediately read upon injection. The average luminescence value of a negative control group (PBS-treated) was subtracted from all other read luminescence values to take into account the background. Finally, each dose–response curve was fit using the GraphPad Prism software (log(agonist) vs response–four parameter fit) to estimate EC_50_ values.

### Gene Expression in BMDCs and Cancer Cell Lines

Relative gene expression of *Ifnb1* (Mm00445235_s1), *Tnf* (Mm00443258_m1), *Cxcl10* (Mm00445235_m1), and/or *IL12* (Mm00434174_m1) in BMDCs, B16.F10 melanoma cells, and EO771 breast cancer cells was quantified by RT-qPCR following treatment with SLR-LNP or controls. In brief, 1,000,000 μ_B_DCs/well, 500,000 B16.F10 cells/well, or 500,000 EO771 cells/well were seeded in a 12-well plate and treated with PBS, empty LNP cSLR-LNP, or SLR-LNP for 24 h. RNA was isolated using an RNeasy Mini kit (Qiagen, Germantown, MD) per manufacturer’s instructions. cDNA synthesis was performed with an iScript kit (Bio-Rad) to reverse-transcribe 1 μg of isolated RNA per sample, and RT-qPCR was performed using a TaqMan Mastermix kit (Thermo Fisher Scientific) with *Hmbs* (Mm01143545_m1) used as a housekeeping gene. The results were then analyzed via the ΔΔCt method.

### Evaluation of BMDC Activation

BMDC activation was evaluated by flow cytometric analysis of the surface CD80, CD86, and MHC-II expression. Briefly, 1,000,000 μ_B_DCs/well were seeded in 12-well plates and treated with PBS, empty LNP, cSLR-LNP, or SLR-LNP for 24 h. Cells were collected and washed with 1% BSA in PBS and stained with FITC-anti-CD11c (1:100), APC/Cy7-anti-MHC class II (1:100), PE-anti-CD86 (1:100), and APC-anti-CD80 (1:100) (Biolegend) antibodies and DAPI (live/dead) stain (1:20,000). Cells were analyzed by using a CellStream flow cytometer (Luminex).

### Animal Ethics Statement

Studies using mice were conducted under an Animal Care Protocol approved by the Vanderbilt University Institutional Animal Care and Use Committee (VU IACUC). Health assessments of animals were completed using standard operating procedures approved by the VU IACUC.

### Subcutaneous Single B16–F10 Tumor Model

B16–F10 (3 × 10^5^) cells were injected subcutaneously into the right flank of female 6- to 7-week-old C57BL/6 mice (The Jackson Laboratory). B16–F10 (40–60 mm^3^) tumors were treated intratumorally with vehicle (PBS), empty LNP, cSLR-LNP (10 μg), and SLR-LNP (10 μg) in 50 μL. For evaluation of gene expression via qPCR, mice were treated once intratumorally, and mice were euthanized 24 h postinjection. For evaluation of therapeutic efficacy, mice were administered SLR-LNPs or controls intratumorally every 3 days for 3 total injections. Tumor volume (*V*_tumor_) measurements were taken 3x weekly using calipers, and the volume was calculated using (*V*_tumor_ = *L* × *W*^2^ × 0.5). When subcutaneous tumors reached >1500 mm^3^ according to the above calculation, the mice bearing the large tumors were euthanized.

### Orthotopic EO771 Breast Tumor Model

EO771 (2.5 × 10^5^) cells were injected into the left inguinal mammary fat pad of female 6- to 7-week-old C57BL/6 mice (The Jackson Laboratory, Bar Harbor, ME). Mice were randomized into treatment groups, and mice were intravenously administered vehicle (PBS), empty LNP, cSLR-LNP (10 μg), or SLR-LNP (10 μg) in 100 μL of PBS 3 times spaced 3 days apart. In studies evaluating effects on the TME by qRT-PCR, Western blot analysis, or flow cytometry, mice were euthanized 24 h after the last treatment. In studies investigating combination effects with ICIs (αPD-1) or T-cell depletion antibodies (αCD4 or αCD8), mice received intraperitoneal injections of 100 μg of antibody in dilution buffer every 3 days for 5 total injections. For T-cell depletion studies, antibody treatment began 24 h before treatment with SLR-LNPs. Tumor volume (*V*_tumor_) measurements were taken 3× weekly using calipers, and the volume was calculated using (*V*_tumor_ = *L* × *W*^2^ × 0.5). When mammary fat pad tumors reached >1500 mm^3^ according to the above calculation, the mice bearing the large tumors were euthanized.

### In Vivo Imaging of Interferon Response

B16.F10 melanoma cells were transduced with the Cignal Lenti Reporter construct (Qiagen) to express luciferase in an ISRE-dependent as described previously.^52^ Six- to 8-week-old C57BL/6 mice (The Jackson Laboratory) were anesthetized with isoflurane, and their right dorsal flanks were shaved. Mice were inoculated with 1 × 10^6^ B16.F10 interferon reporter cells in 100 μL of PBS. When tumors reached ∼100 mm^3^, the mice received a 50 μL IT injection of either PBS or SLR-LNP at a dose corresponding to 10 μg of SLR20. At each time point (0 and 6 h), mice were subcutaneously injected with 150 μL of 30 mg/mL D-luciferin (Thermo Fisher Scientific) in PBS. After 15 min, luminescence images were captured using an IVIS Lumina III (PerkinElmer). Relative IFN production for each tumor was calculated at 6h as a fold-change relative to the respective *t* = 0 h value for each mouse.

### Subcutaneous B16-OVA Two Tumor Model

Ovalbumin-expressing B16–F10 melanoma (B16-OVA) cells (2.5 × 10^5^) were subcutaneously injected into the right and left flank regions of 6- to 7-week-old female C57BL/6 mice (The Jackson Laboratory). Right flank B16-OVA tumors were treated intratumorally with vehicle (PBS), empty LNP, cSLR-LNP, or SLR-LNP (10 μg) every 3 days for 3 total injections, beginning on day 8 when tumors reached ∼100 mm^3^. Mice in groups receiving αPD-1 and αCTLA-4 (100 μg, every 3 days for 5 injections, beginning on day 8, BioXcell) were treated intraperitoneally. Tumor volume (*V*_tumor_) measurements were taken 3× weekly using calipers, and the volume was calculated using (*V*_tumor_ = *L* × *W*^2^ × 0.5). When mammary fat pad tumors reached >1500 mm^3^ according to the above calculation, the mice bearing the large tumors were euthanized.

### Lung Metastatic B16–F10 Tumor Model

Six- to 8-week-old C57BL/6 mice (The Jackson Laboratory) were administered a single intravenous injection of 0.5 × 10^6^ luciferase-expressing B16.F10 cells (B16-LUC) suspended in PBS via the tail vein. On day 3 post-tumor inoculation, mice were treated intravenously with PBS, cSLR-LNP (10 μg), or SLR-LNP (10 μg) every 3 days for 3 doses total. Mice in groups receiving αPD-1 and αCTLA-4 (100 μg, every 3 days for 4 doses, BioXcell) were injected intraperitoneally. Twenty days post-tumor inoculation, mice were euthanized, and lungs were excised. Lungs were weighed and imaged. Lungs were then placed in black 12-well plates (Cellvis) and incubated in 1 mg/mL Pierce D-Luciferin, Monopotassium Salt (Thermo Fisher Scientific) reconstituted in PBS, and luminescence images were captured 5 m thereafter on the IVIS Lumina III (PerkinElmer). The luminescence was quantified as total radiant flux (p/s) for each set of lungs.

### qRT-PCR of Tumor Tissue

C57BL/6 mice bearing either subcutaneous B16–F10 or orthotopic EO771 breast tumors were treated with SLR-LNPs or controls as described above and euthanized 6 or 24 h later. Tumors were harvested, snap-frozen in liquid nitrogen, and stored at −80 °C prior to analysis. Tumors were homogenized in RLT lysis buffer using a TissueLyser II (Qiagen), and RNA was isolated by an RNeasy mini kit (Qiagen). An iScript cDNA synthesis kit (Bio-Rad) was used to reverse-transcribe 1 μg of isolated RNA per sample. Then, RT-qPCR was performed using a TaqMan Mastermix kit (Thermo Fisher Scientific), with *Hmbs* (Mm01143545_m1) used as a housekeeping gene. Relative gene expression of *Ifnb1* (Mm00445235_s1), *Tnf* (Mm00443258_m1), *Cxcl10* (Mm00445235_m1), and/or *IL12* (Mm00434174_m1) were measured, and the results were then analyzed via the ΔΔCt method.

### Western Blot Analysis of EO771 Tumors

Female C57BL/6 mice with 100–200 mm^3^ EO771 tumors in the mammary fat pad were treated as described earlier with SLR-LNP or the controls. Mice were euthanized at 24 h following the last injection and tissues, were snap-frozen until analysis. EO771 tumors in RIPA lysis buffer (Santa Cruz) were homogenized using a TissueLyser II (Qiagen). The total protein concentration in the lysate was quantified using a BCA assay (Thermo Scientific, Waltham, Massachusetts). Samples were diluted to the same concentration and boiled with loading buffer at 95 °C for 10 min. Then, these samples were run on an SDS-PAGE to separate the proteins by molecular weight, and they were transferred to a nitrocellulose membrane via a semidry transfer system (Bio-Rad Laboratories, Hercules, California). The nitrocellulose membranes were then washed, blocked with milk for 1 h, and incubated with primary α-mouse antibodies (pIRF3, IRF3, RIG-I, and β-actin) at 4 °C overnight. The next day, the blots were again washed, and they were then incubated with HRP-conjugated secondary antibodies (Promega). Protein bands were imaged on a ChemiDoc XRS+system (Bio-Rad) using an immobile Western Chemiluminescent HRP Substrate Kit (Millipore Sigma). The blots were analyzed using ImageJ. β-actin was used as a loading control for normalization of samples.

### Flow Cytometric Analysis of EO771 Tumors

Female C57BL/6 mice with 100–200 mm^3^ EO771 tumors in the mammary fat pad were treated with SLR-LNPs (10 μg, via tail vein injection) or PBS every 3 days for 3 total injections. Mice were euthanized 24 h after the last injection. Their tumors were harvested and weighed. Then, these tumors were digested using digestion in RPMI 1640 containing 125 μg/mL deoxyribonuclease I and 500 μg/mL collagenase III for 30 min at 37 °C and an OctoMACS separator (Miltenyi) to achieve a cell suspension. This suspension was strained (70 μm) to achieve a single-cell suspension. These cells were then pelleted and resuspended in ACK lysis buffer (Gibco) to lyse any red blood cells in the pellet. Then, the pellet was washed with PBS and resuspended in a flow buffer (5% BSA + 0.1% dasatinib in PBS). For analysis of T cells, the cells were stained with APC-αCD3 (17A2), APC/Cy7-αCD4 (RM4-5), PE-αCD8α (53–6.7), PE/Cy7-αCD45 (30-F11), and DAPI. For analysis of myeloid and NK cells, the cells were stained with APC-αCD45 (30/F11), PerCP/Cy5.5-αCD11b (M1/70), PE/Cy7-αF4/80 (BM8), Alex Flour 488-αCD11c (N418), APC/Cy7-αMHC-II (M5/114.15.2), PE-αNK1.1 (PK136), BV605-Gr-1 (1A8), and DAPI. After staining, the cells were washed with a flow buffer two times and resuspended in the flow buffer containing AccuCheck counting beads. The samples were then run on a BD LSR II flow cytometer and analyzed using FlowJo (ver. 10; Tree Star). Representative flow cytometry plots and gating schemes are shown in Figure S7.

### Histology

Major organs (liver, spleen, kidney, lung, brain, heart, pancreas, and bone marrow (sternum)) were isolated 24 h after treatment with 10 μg SLR-LNP or relevant control, and the organs were routinely fixed in 10% neutral buffered formalin. They were then embedded in paraffin, sectioned, and H&E-stained by the Vanderbilt Translational Pathology Shared Resource for blinded evaluation by a board-certified veterinary pathologist.

### Biodistribution and Pharmacokinetics

Female C57BL/6 mice bearing 100–200 mm^3^ EO771 mammary fat pad tumors were administered a single 100 μL intravenous injection of PBS, SLR-AF647-LNP (containing 1:5 AF647-labeled-cSLR:cSLR) or DiR-LNP (containing 0.1% DiR) at a dose corresponding to 10 μg SLR-LNP (*n* = 5 per treatment group/time point). Mice were euthanized at 1, 4, and 24 h posttreatment. Organs were excised, and fluorescence (i.e., radiant efficiency) was measured on the IVIS Lumina III (PerkinElmer). The excitation/emission filter pairs used to quantify radiant efficiency were 640 nm/710 nm for the organs of the mice treated with SLR-AF647-LNPs and 720 nm/790 nm for the organs of the mice treated with DiR-LNPs. To determine the pharmacokinetics of the LNPs, healthy C57BL/6 mice were intravenously injected with 100 μL of SLR-Cy5-LNP (containing 1:2 Cy5-labeled-cSLR:cSLR) at a dose corresponding to 10 μg of SLR-LNP (*n* = 5). Blood was collected using heparinized capillary tubes (DWK Life Sciences) at discrete time points. One microliter of blood from each tube was mixed with 50 μL of PBS, and the diluted plasma was collected for analysis. The amount of Cy5 was determined by fluorescence spectroscopy using a plate reader (645 nm/675 nm). Pharmacokinetic analysis was performed in GraphPad Prism using a one-phase decay.

### Statistical Analysis

Data were plotted using Prism 9 (GraphPad) as the mean ± SEM unless noted in the figure legend. Data were analyzed via Student’s *t* test or a one-way ANOVA followed by Tukey’s adjustment for multiple comparisons. A log-rank test was used to compare Kaplan–Meier survival data. *P* values < 0.05 were considered statistically significant in all studies.

## Supporting Information Available

Detailed DLin-MC3-DMA (MC3) synthesis and characterization, in vitro delivery of SLR-LNP to bone marrow-derived dendritic cells and tumor cells, evaluation of systemic cytokine response to intravenously administered SLR-LNP, evaluation of systemic toxicity of intravenously administered SLR-LNP (including histology of major clearance organs, biodistribution and pharmacokinetic data, and dot plots representative of the flow cytometry gating strategy related to Figure 5B (PDF)

## Notes

A preprint version of this manuscript is accessible via: L.W.-B.; M.W.; L.E.P.; J.Y.; K.M.G.; K.W.B.; C.S.C.; K.N.G.-C.; D.U.; V.K.; O.F.; A.R.; A.M.P.; J.T.W. A Nanoparticle RIG-I Agonist for Cancer Immunotherapy. 2023, 537919. bioRxiv. 10.1101/2023.04.25.537919 (accessed April 25, 2023).
