# Supplementary material for: Nanoparticle Retinoic Acid-Inducible Gene I Agonist for Cancer Immunotherapy
Source: ACS Nano. 2024 Apr 23;18(18):11631–43. doi: 10.1021/acsnano.3c06225 (PMC11080455; doi:10.1021/acsnano.3c06225)
Supplement: Supplementary file 1 — nn3c06225_si_001.pdf [file nn3c06225_si_001.pdf]

SUPPORTING INFORMATION:

A Nanoparticle Retinoic Acid-inducible Gene I Agonist for Cancer Immunotherapy

Lihong Wang-Bishop[a,^], Mohamed Wehbe[a,^], Lucinda E. Pastora[a,^], Jinming Yang[b,c], Blaise R. Kimmel[a,#], Kyle M. Garland[a], Kyle W. Becker[a], Carcia S. Carson[d], Eric W. Roth[e], Katherine N. Gibson-Corley[f,g], David Ulkoski[h], Venkata Krishnamurthy[h], Olga Fedorova[i,j], Ann Richmond[b,c], Anna Marie Pyle[i,j,k], John T. Wilson[a,d,f,l-p,*]

[a]Department of Chemical and Biomolecular Engineering, Vanderbilt University, Nashville, TN, 37212, USA
[b]Department of Pharmacology, Vanderbilt University Medical Center, Nashville, TN, 37232, USA
[c]Tennessee Valley Healthcare System, Department of Veterans Affairs, Nashville, TN, 37212, USA
[d]Department of Biomedical Engineering, Vanderbilt University, Nashville, TN, 37212, USA
[e]Northwestern University Atomic and Nanoscale Characterization Experimental (NUANCE) Center, Northwestern University, Evanston, IL, 60208, USA
[f]Department of Pathology, Microbiology, and Immunology, Vanderbilt University Medical Center, Nashville, TN, 37232, USA
[g]Department of Medicine, Vanderbilt University Medical Center, Nashville, TN, 37232, USA
[h]Advanced Drug Delivery, Pharmaceutical Sciences, R&D, AstraZeneca, Boston, MA, 02451, USA
[i]Department of Molecular, Cellular and Developmental Biology, Yale University, New Haven, CT, 06520, USA
[j]Howard Hughes Medical Institute, Chevy Chase, MD, 20815, USA
[k]Department of Chemistry, Yale University, New Haven, CT, 06520, USA
[l]Vanderbilt Institute of Chemical Biology, Vanderbilt University, Nashville, TN, 37212, USA
[m]Vanderbilt Institute of Nanoscale Science and Engineering, Vanderbilt University, Nashville, TN, 37212, USA
[n]Vanderbilt Institute for Infection, Immunology, and Inflammation, Vanderbilt University, Nashville, TN, 37212, USA
[o]Vanderbilt Center for Immunobiology, Vanderbilt University Medical Center, Nashville TN, 37232, USA
[p]Vanderbilt Ingram Cancer Center, Nashville, TN, 37232, USA

[^]equally contributing authors
[#]current address: Department of Chemical and Biomolecular Engineering, The Ohio State University, Columbus, OH, 43210, USA
*Corresponding author: john.t.wilson@vanderbilt.edu

Supplementary Methods – DLin-MC3-DMA (MC3) Synthesis

<u>Overall Synthetic Scheme:</u>

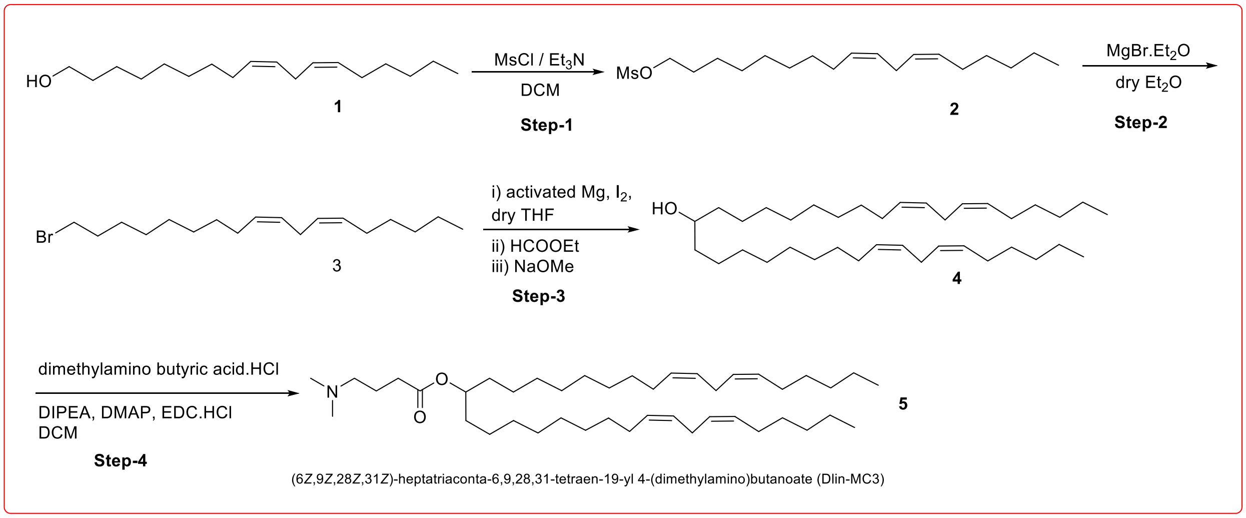

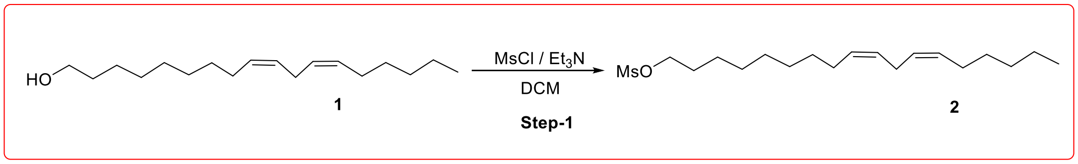

Step-1: (9Z,12Z)-octadeca-9,12-dien-1-yl methanesulfonate

Mesyl-Cl (0.702 ml, 9.01 mmol) was added dropwise to a stirred mixture of (9Z,12Z)-octadeca-9,12-dien-1-ol (2 g, 7.51 mmol) and TEA (4.29 ml, 30.77 mmol) in DCM (20 mL) at 0°C under argon. The resulting mixture was stirred at room temperature for 16 hours. The reaction mixture was diluted with DCM (50 mL) and washed with saturated aqueous NaCl (50 mL). The organic layer was dried over Na2SO4, filtered, and concentrated under reduced pressure to dryness to afford crude product. The resulting residue was purified by flash silica chromatography, elution gradient 0 to 100% EtOAc in hexanes. Product fractions were concentrated under reduced pressure to dryness to afford (9Z,12Z)-octadeca-9,12-dien-1-yl methanesulfonate (1.828 g, 70.7 %) as a colorless oil. ^{1}H NMR (500MHz, CHLOROFORM-d) δ ppm 0.90 (t, *J* = 6.9 Hz, 3H), 1.24 - 1.47 (m, 16H), 1.69 - 1.82 (m, 2H), 2.06 (m, *J* = 7.3 Hz, 4H), 2.78 (t, 2H), 3.01 (s, 3H), 4.23 (t, *J* = 6.6 Hz, 2H), 5.27 - 5.46 (m, 4H).

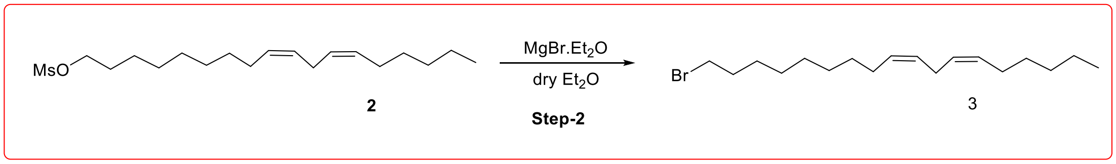

Step-2: (6Z,9Z)-18-bromooctadeca-6,9-diene

To a solution of (9Z,12Z)-octadeca-9,12-dien-1-yl methanesulfonate (1.828 g, 5.31 mmol) in THF (10 mL), lithium bromide (0.921 g, 10.61 mmol) was added in one portion under argon. The resulting clear colorless solution was heated to 65 °C and stirred for 16 hours. The reaction mixture was diluted with MTBE (30 mL) and washed with water (2 x 25 mL). The organic layer was dried (Na2SO4) and concentrated under reduced pressure to give crude product. The resulting residue was purified by flash silica chromatography, elution gradient 0 to 100% EtOAc in hexanes. Product fractions were concentrated under reduced pressure to dryness to afford (6Z,9Z)-18-bromooctadeca-6,9-diene (1.571 g, 90 %) as a colorless oil. 1H NMR (500MHz, CHLOROFORM-d) δ ppm 0.90 (t, J = 6.9 Hz, 3H), 1.24 - 1.49 (m, 16H), 1.87 (m, 2H), 2.06 (t, J = 7.0 Hz, 4H), 2.79 (s, 2H), 3.42 (t, J = 6.9 Hz, 2H), 5.26 - 5.48 (m, 4H).

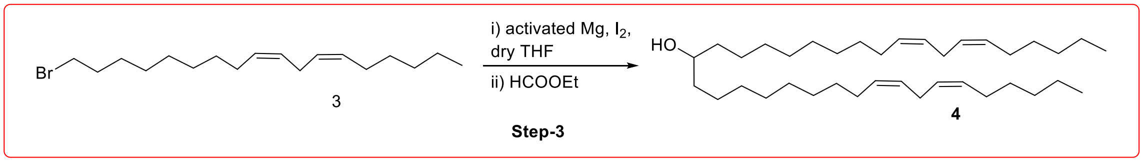

Step-3: (6Z,9Z,28Z,31Z)-heptatriaconta-6,9,28,31-tetraen-19-ol

Mg turnings (1.5 g, 0.0625 mole, 1.12 eq) and two crystals of iodine were taken in to clean dry 250 mL 2 neck round bottom flask. (6Z,9Z)-18-bromooctadeca-6,9-diene (1.34 g, 0.0558 mole, 1 eq) dissolved in 50 mL of THF was added slowly dropwise at room temperature. Exothermic conditions were observed. After completion of the addition, reaction mixture was heated at 50°C for 5h. The progress of the reaction was monitored by TLC. Reaction mixture was slowly allowed to come at 0°C and ethyl formate (4.26 g, 0.0575 mole, 1.03 eq) dissolved in 10 mL of THF was added drop wise at 0°C. Reaction mixture was slowly allowed to come at RT and continued for overnight with proper stirring, the reaction was monitored by TLC. The reaction mixture was then cooled to 0°C and quenched with 2 M HCl solution (100 mL). The reaction the mixture was diluted with ethyl acetate (100 mL) and separated. The aqueous layer was extracted with ethyl acetate (3 x 50 mL) and the combined organic layers were washed with brine solution (1 x 50 mL). The organics were dried over Na_2SO_4, filtered, and concentrated under reduced pressure to obtained light yellow oil. The compound was purified by flash column chromatography (silica gel, 0-10% Ethyl acetate in hexanes) and the pure product fractions were evaporated to afford (6Z,9Z,28Z,31Z)-heptatriaconta-6,9,28,31-tetraen-19-ol (1.4 g, 60%) as yellow oil. 1H NMR (500MHz, CHLOROFORM-d) δ ppm 0.87 – 0.91 (t, 6H), 1.20 - 1.43 (m, 46H), 2.01 – 2.06 (m, 9H), 2.75 – 2.79 (t, 4H), 3.58 (m, 1H), 5.29 – 5.43 (m, 8H).

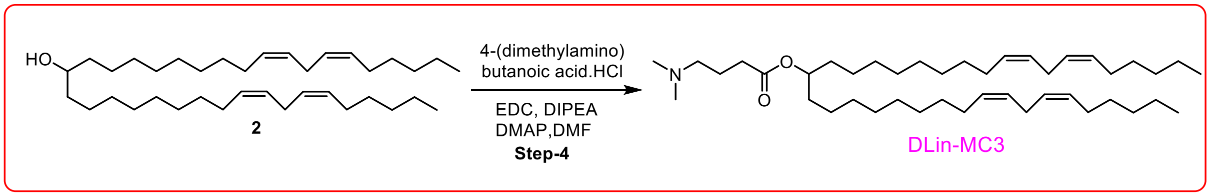

Step-4:(6Z,9Z,28Z,31Z)-heptatriaconta-6,9,28,31-tetraen-19-yl 4 (dimethylamino)butanoate (DLin-MC3-DMA)

Dimethylaminobutyric acid hydrochloride (0.950 g, 0.00567 mole, 2 eq) was added in one portion to (6Z,9Z,28Z,31Z)-heptatriaconta-6,9,28,31-tetraen-19-ol (1.5 g, 0.00284 mole, 1 eq) was dissolved in DMF (50 mL). The reaction mixture was cooled to 0°C and diisopropylethylamine (58 mL, 0.3308mole, 5 eq) and DMAP (1.61 g, 0.01323 mole, 0.2 eq) were added. After stirring for 5 min, EDC (2.37 g, 0.00567 mole, 2 eq) was added at 0°C The reaction was continued at room temperature for 16 h and monitored by TLC. The reaction mixture was cooled to 0°C and quenched with 10% citric acid solution (50 mL). The aqueous solution was extracted with DCM (3 x 50 mL) and the combined organic layers were washed with brine solution (50 mL). The organic layer was dried over Na_2SO_4, filtered, and concentrated under reduced pressure to obtained crude oil compound. The compound was purified by flash column chromatography (silica gel, 0-40% [20% MeOH and 1% NH4OH in DCM] in DCM). Product fractions were concentrated under reduced pressure to dryness to afford (6Z,9Z,28Z,31Z)-heptatriaconta-6,9,28,31-tetraen-19-yl 4-(dimethylamino)butanoate (1.42 g, 78 %) as a pale yellow oil. 1H NMR (500MHz, CHLOROFORM-d) δ ppm 0.89 (t, J = 6.8 Hz, 6H), 1.33 (m, 44H), 1.53 (d, 6.2 Hz, 4H), 2.04 (m, 11H), 2.53 (t, 6.4 Hz, 2H), 2.77 (t, J = 6.6 Hz, 4H), 2.80 (s, 2H), 3.00 (s, 6H), 3.26 (t, 7.7 Hz, 2H), 4.85 (m, 1H), 5.36 (m, 8H).

Supplementary Data:

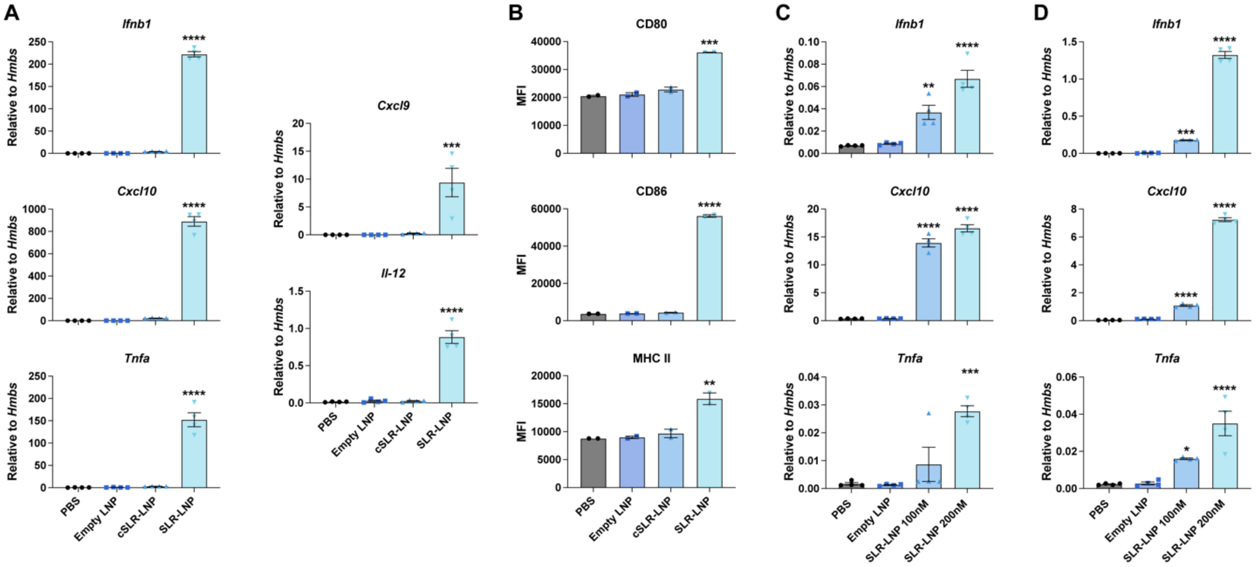

Figure S1: Lipid nanoparticle delivery of SLR20 activates RIG-I in immune and tumor cells. (A) RT-qPCR analysis of bone marrow derived dendritic cells (BMDCs) treated with indicated LNP formulation or PBS for 24h. (n=4, *** $p<0.001$, ****$p<0.0001$, by one-way ANOVA). **(B)** Flow cytometric quantification (median fluorescent intensity) of CD80, CD86, and MHC II expression on BMDCs stained after 24h treatment with indicated LNP formulation or PBS. (n=2, **$p<0.01$, *** $p<0.001$, ****$p<0.0001$, by one-way ANOVA). RT-qPCR analysis of **(C)** B16.F10 melanoma cells and **(D)** EO771 breast cancer cells treated with indicated LNP formulation or PBS for 24h. (n=4, *$p<0.05$, **$p<0.01$, *** $p<0.001$, ****$p<0.0001$, by one-way ANOVA). All statistical data are presented as mean ± SD.

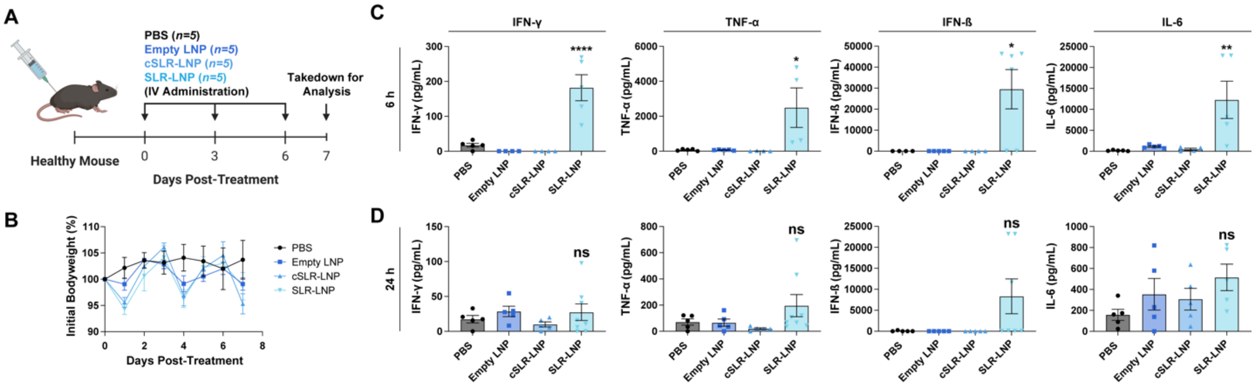

Figure S2. Evaluation of systemic cytokine response to intravenously administered SLR/LNPs. (A) Schematic diagram showing the treatment schedule of healthy mice treated with 10 μg/mL SLR-LNP or control. **(B)** Body weight change of the mice after designated treatments. Quantification of plasma cytokines **(C)** 6 hours and **(D)** 24 hours after the final treatment (n=3-5, *P ≤ 0.05, **P ≤ 0.01, ***P ≤ 0.001 by one-way ANOVA).

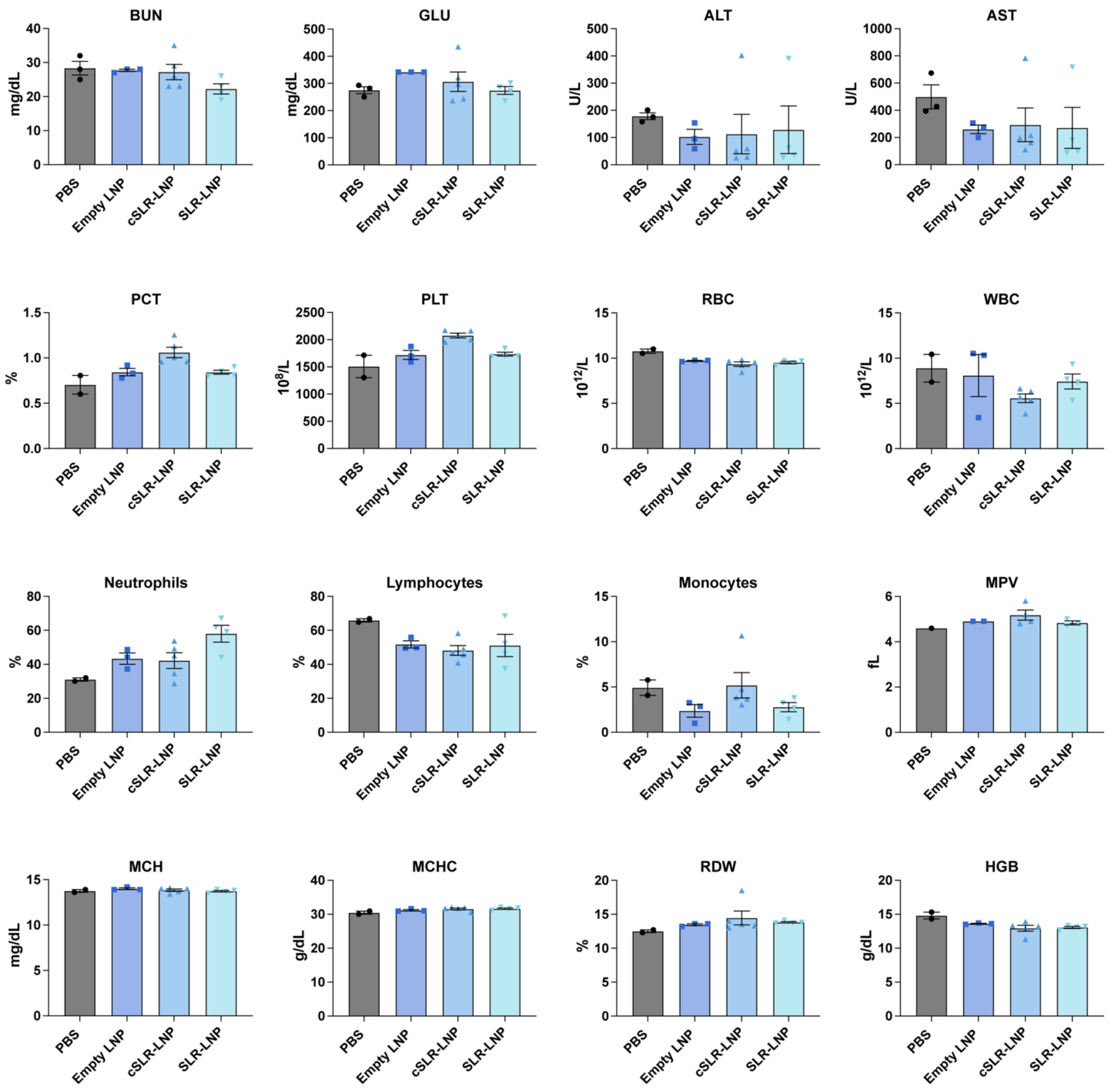

Figure S3. Evaluation of toxicity of SLR-LNPs in mice. Blood biochemistry of healthy non-tumor-bearing syngeneic C57BL/6 mice. Animals (n=3-5) were administered 3 treatments and sacrificed 24 hours after final the final treatment. Blood samples were collected and analyzed to determine changes in RBCs, white blood cells (WBCs), neutrophils, platelets, and lymphocytes. Serum samples were used to analyze liver and kidney function, by measuring changes in ALT/AST, blood urea nitrogen (BUN), and creatinine. No significant changes were observed. (*P ≤ 0.05, **P ≤ 0.01 compared to PBS by one-way ANOVA).

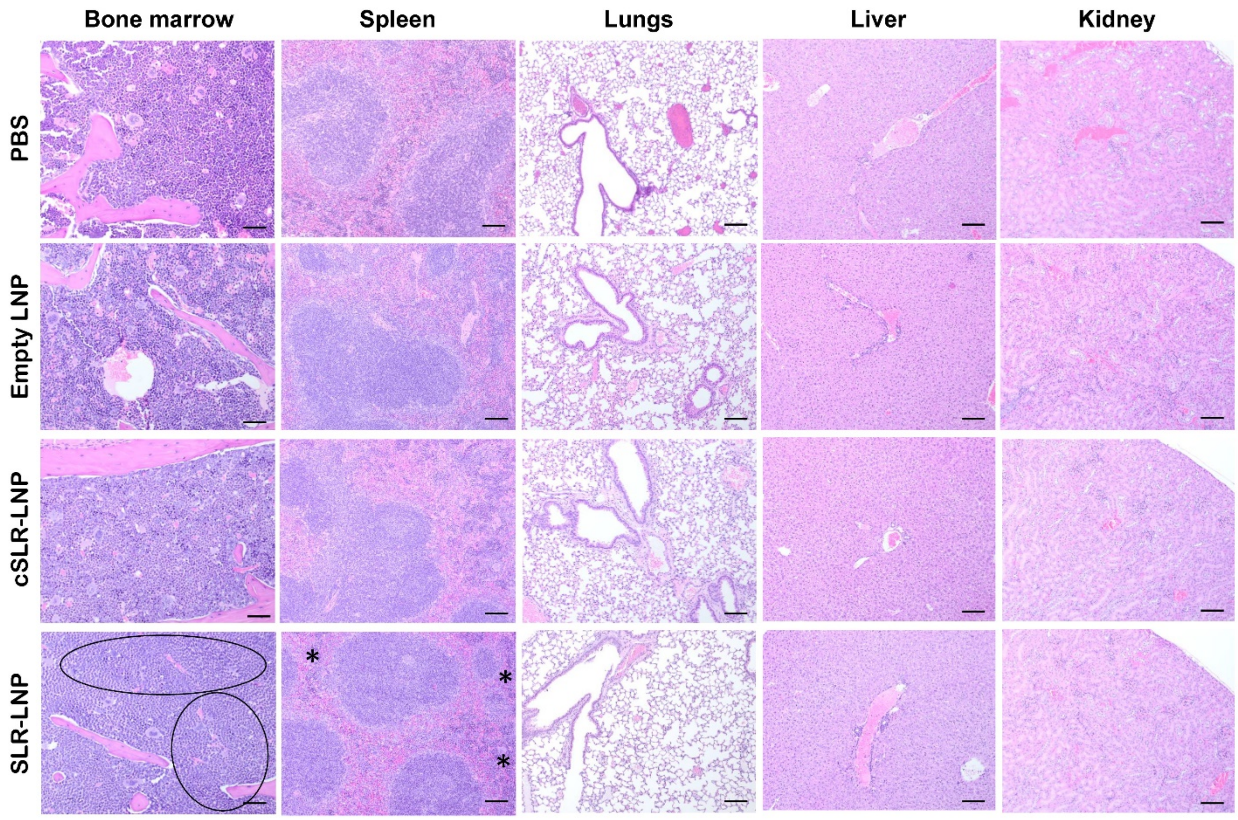

Figure S4. Histopathology. Representative photomicrographs of H&E-stained slides of the bone marrow, spleen, lung, liver, and kidneys from mice treated with PBS (top row), empty LNP, cSLR-LNP, and SLR-LNP (bottom row). Encircled areas identify an increased ratio of myeloid to erythroid bone marrow precursor cells while * indicate extramedullary hematopoiesis. Scale bars = 100um for spleen, lungs, liver, and kidney images while scale bars = 50um for bone marrow images.

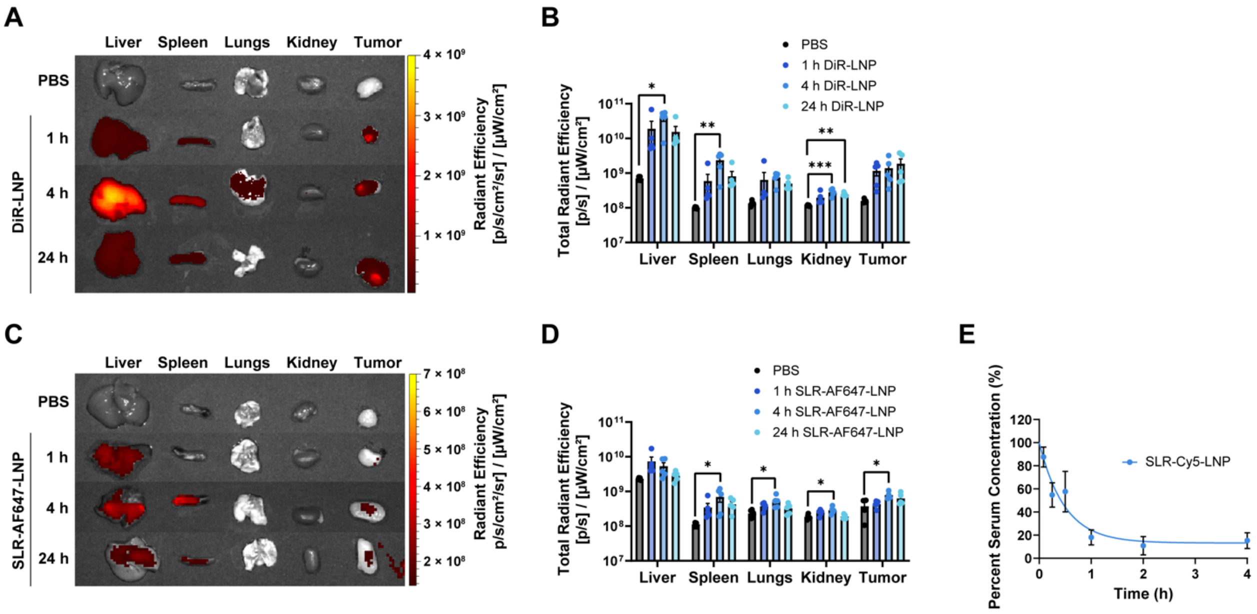

Figure S5. Biodistribution and Pharmacokinetics. **(A)** Representative IVIS fluorescent images of excised major organs and orthotopic EO771 tumors and **(B)** quantification of average radiant efficiencies 1, 4, and 24 h following intravenous administration of vehicle (PBS) or DiR-LNP (n=4-5). *P* values determined by one-way ANOVA with post-hoc Tukey's correction for multiple comparisons; **P*≤0.05, ***P*≤0.01, and ****P*≤0.001. **(C)** Representative IVIS fluorescent images of excised major organs and orthotopic EO771 tumors and **(D)** quantification of average radiant efficiencies 1, 4, and 24 h following intravenous administration of vehicle (PBS) or SLR-AF647-LNP (n=4-5). *P* values determined by one-way ANOVA with post-hoc Tukey's correction for multiple comparisons; **P*≤0.05. **(E)** Pharmacokinetics of SLR-Cy5-LNP injected at 10 µg/mL in healthy female C57BL/6 mice (n=5).

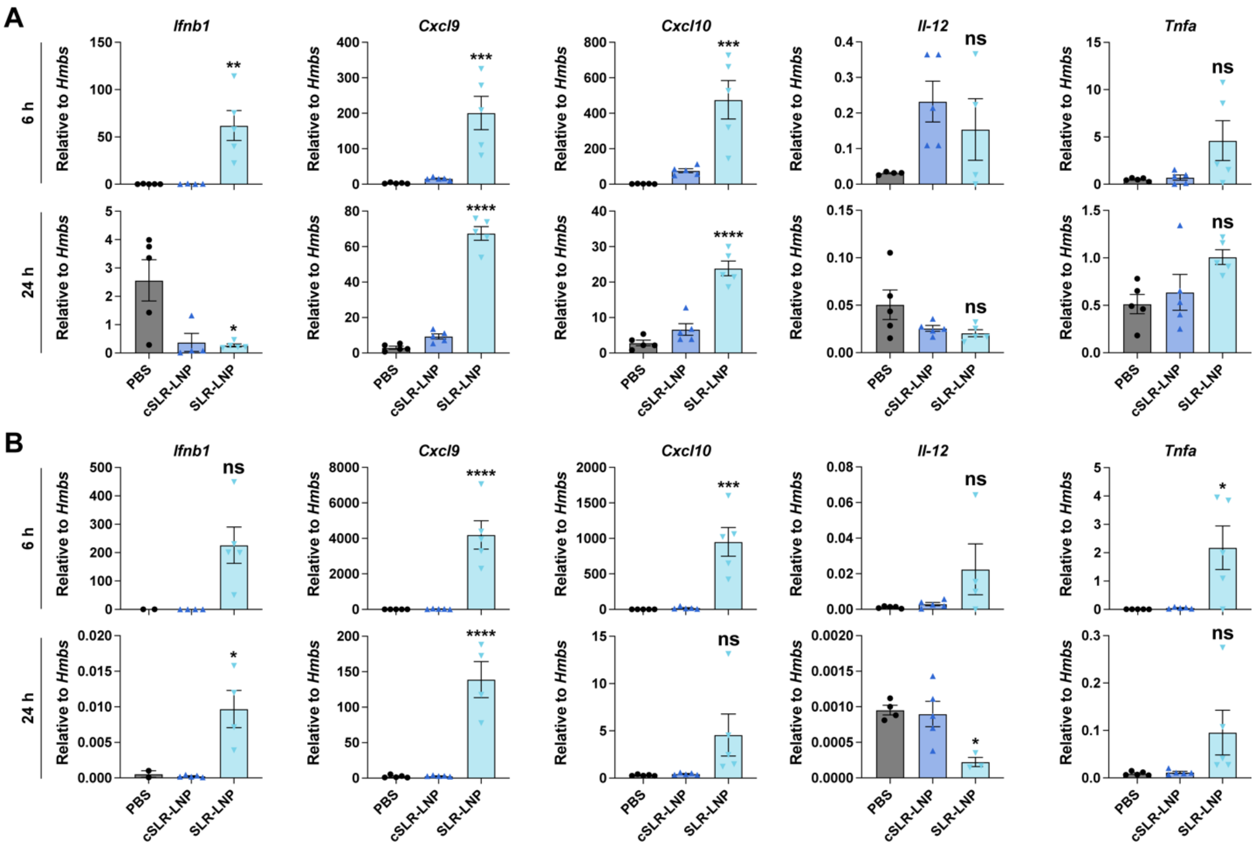

Figure S6. Systemically administered SLR-LNPs activate RIG-I in liver and spleen. qRT-PCR analysis of **(A)** spleen and **(B)** liver 6 or 24 h following a single intravenous injection of SLR-LNP, cSLR-LNP, or PBS (*P≤0.05; **P≤0.01; ***P≤0.001; ****P≤0.0001 vs. PBS by one-way ANOVA with post hoc Tukey's correction for multiple comparisons; n=5 mice per group).

1H NMR:

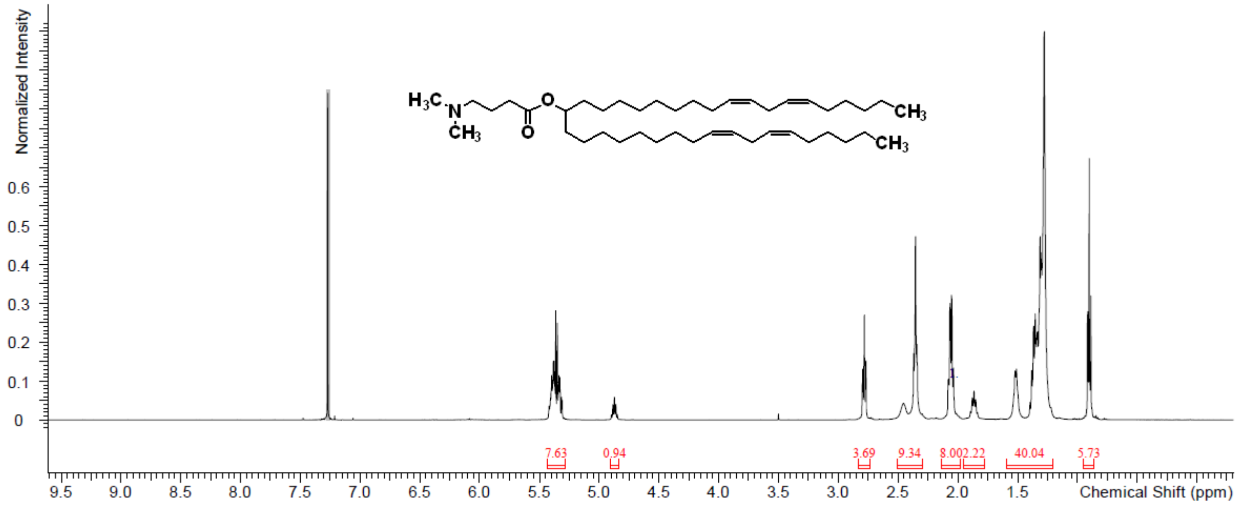

UPLC-ELSD:

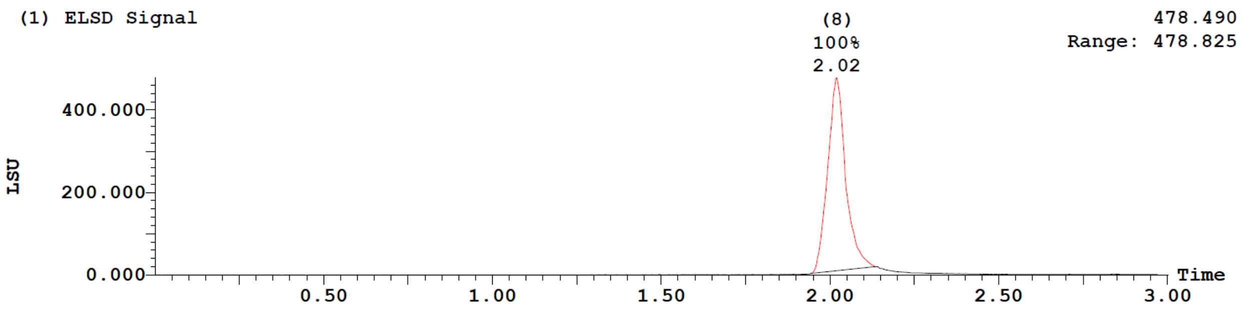

Peak Number	Compound	Time	AreaAbs	Area %Total	Width	Height	Mass Found
8		2.02	3e+004	100.00	0	5e+005	Not Found

MS:

Peak ID	Compound	Time	Mass Found
8		2.04	Not Found

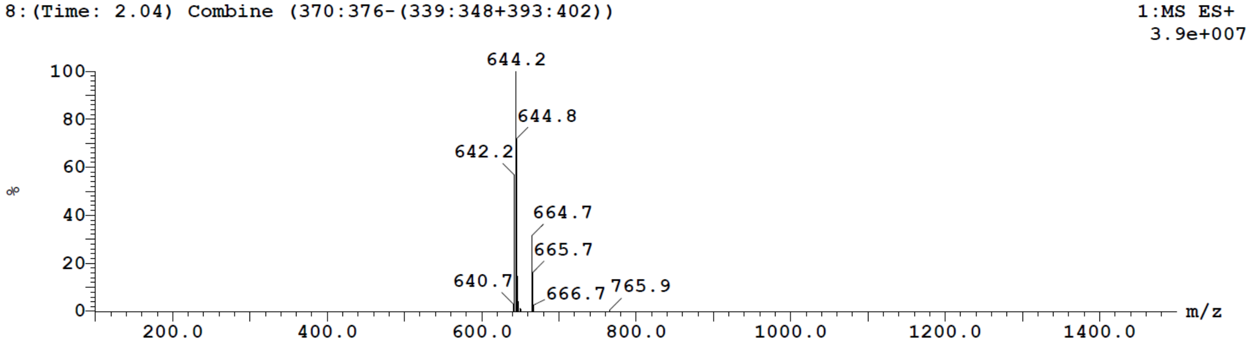

Figure S7: Chemical Characterization of DLin-MC3-DMA.

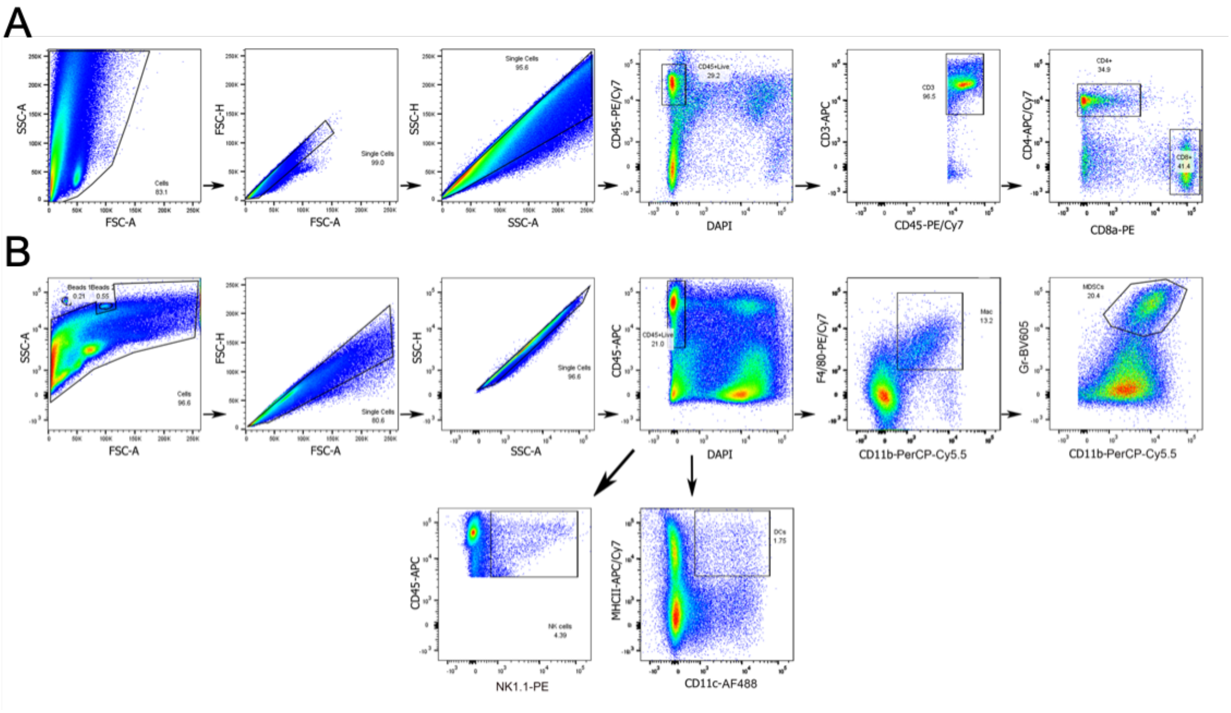

Figure S8: Representative flow cytometry dot plots showing gating strategy related to Figure 5B for analysis of tumor infiltrating **(A)** $CD4^+$ and $CD8^+$ T cells and **(B)** macrophages ($CD11b^+F4/80^+$), natural killer cells (NK 1.1^+), dendritic cells ($CD11c^+MHCII^+$), and MDSCs ($CD11b^+Gr\text{-}1^+$).
